# Differential immunogenicity in people living with HIV with varying CD4 levels after bivalent mRNA COVID-19 booster vaccination

**DOI:** 10.1371/journal.pone.0317940

**Published:** 2025-04-29

**Authors:** Napon Hiranburana, Nattakarn Thippamom, Anchalee Avihingsanon, Supaporn Wacharapluesadee, Sasiwimol Ubolyam, Stephen J. Kerr, Chee Wah Tan, Lin-Fa Wang, Opass Putcharoen

**Affiliations:** 1 Division of Infectious Diseases, Department of Medicine, Faculty of Medicine, Chulalongkorn University, Bangkok, Thailand; 2 HIV-NAT, Thai Red Cross AIDS and Infectious Diseases Reserach Centre, Bangkok, Thailand; 3 Faculty of Medicine, Center of Excellence in Tuberculosis, Chulalongkorn University, Bangkok, Thailand; 4 Thai Red Cross Emerging Infectious Diseases Clinical Center, King Chulalongkorn Memorial Hospital, Bangkok, Thailand; 5 Faculty of Medicine, Biostatistics Excellence Centre, Chulalongkorn University, Bangkok, Thailand; 6 The Kirby Institute, University of New South Wales, Sydney, Australia; 7 Department of Microbiology and Immunology, Infectious Diseases Translational Research Programme, Yong Loo Lin School of Medicine, National University of Singapore, Singapore; 8 Programme in Emerging Infectious Diseases, Duke-NUS Medical School, Singapore; Centers for Disease Control and Prevention, UNITED STATES OF AMERICA

## Abstract

People living with HIV (PLWH) exhibit heterogeneous immune responses, influenced by varying degrees of immune deficiency and viral suppression. The efficacy of COVID-19 vaccines in this population remains underexplored, particularly in those with low CD4 counts. This study assessed the antibody response in PLWH with CD4 T-cell levels ≤200 cells/mm^3^ compared to those with higher levels, following a bivalent mRNA COVID-19 vaccine booster. All participants were on effective Antiretroviral therapy (ART) with virologic suppression and had received the booster more than a year after their last vaccination. Participants with lower CD4 counts exhibited reduced baseline Anti-RBD IgG titers. However, significant increases in Anti-RBD IgG and surrogate virus neutralization test (sVNT) levels were observed across both CD4 groups post-booster. Despite lower Geometric mean titer (GMT) in the low CD4 group, this group achieved a higher post-boost Anti-RBD IgG Geometric mean ratio (GMR), indicating a robust vaccine response facilitated by effective ART. Nonetheless, cross-neutralization against the circulating XBB variant was limited. Although individuals in the low CD4 group exhibited reduced protection at baseline, they showed significant increase in Anti-RBD IgG and sVNT levels across all CD4 counts after receiving the bivalent COVID vaccine booster.

## Introduction

Immunosuppressed individuals, including solid organ transplant recipients, cancer chemotherapy patients, and people with HIV (PLWH) with low CD4 counts, face a heightened risk of severe COVID-19 and mortality [1–3]. While primary COVID-19 vaccination is a key preventive measure, its effectiveness can be compromised in these populations, particularly in those with very low CD4 counts, primarily due to broader impaired antibody response, including a lower affinity of Anti-Receptor-Binding Domain Immunoglobulin G (Anti-RBD IgG), which is the dominant fraction of SARS-CoV-2 antibodies following infection and immunization, to neutralize the virus [4–8].

In PLWH, the immune response to primary mRNA COVID-19 vaccination is closely related to the quantity of CD4 T lymphocytes. In a cohort of PLWH vaccinated with BNT162b2 or mRNA-1273, the lowest humoral and cell-mediated immune responses were observed in those with CD4 cell counts <200 cells/mm^3^. Immune responses were higher in those with CD4 cell counts between 200–500 cells/mm^3^, and highest in individuals with CD4 cell counts ≥500 cells/mm^3^ and HIV-negative controls [5]. This correlation may reflect the effectiveness of HIV control and the state of T-helper cells functionality. Specifically, low CD4 counts might be associated with T-cell exhaustion or hyperactivation, which could impair Antigen presentation by Antigen-presenting cells (APCs) and disrupt B cell dysfunction, leading to a reduced antibody response [9].

The development of a bivalent mRNA vaccine, targeting both the original SARS-CoV-2 strain and the BA.4/BA.5 subvariants showed no statistically significant difference in neutralizing antibodies levels between recipients of the monovalent and bivalent booster as a fourth dose in Phase 2 studies [10,11]. However, these studies were limited by small sample sizes and the exclusion of immunocompromised individuals. Subsequent research indicated increased titers of neutralizing antibodies against various Omicron subvariants (BA.1, BA.5, BQ.1.1, BN.1, and XBB.1), though data on PLWH remains scarce [12,13].

Therefore, this study aimed to assess the antibody response following bivalent mRNA COVID-19 vaccine boosters in HIV-infected individuals with CD4 T lymphocyte counts ≤ 200 cells/mm^3^ compared to those with counts > 200 cells/mm^3^. The study measured responses though overall immunoglobulin levels (Anti-RBD IgG) and SARS-CoV-2 surrogate virus neutralization test (sVNT) against the Omicron variants (XBB1.16 and XBB1.5) circulating in Thailand at the time, as reported by GISAID [14]. Additionally, the study explored the correlation between overall immunoglobulin levels and neutralization capacity, along with other factors influencing the immune response in PLWH.

## Materials and methods

### Study design and participants

This prospective cohort study was conducted at King Chulalongkorn Memorial Hospital and the HIV Netherlands Australia Thailand Research Collaboration (HIV-NAT) in Thailand from July 2023 to March 2024. The study included PLWH aged ≥18 years who were on stable antiretroviral regimen for at least three months and had received at least two doses of primary COVID-19 vaccine more than three months before enrolment. Exclusion criteria included virologic failure, defined as an HIV viral load exceeding 200 copies/mL and high-level immunosuppression, such as the use of prednisolone at doses exceeding 20 mg/day, methotrexate at doses exceeding 0.4 mg/kg/week, administration of rituximab or TNF-alpha blockers within the past year, active autoimmune disease, or prior treatment with monoclonal antibodies or JAK inhibitors. Participants were also excluded if they had contraindications to COVID-19 vaccination, had received convalescent plasma for COVID-19, had received any other vaccination within 15 days prior to enrolment, or had a self-reported history of SARS-CoV-2 infection within the 3 months prior to enrolment.

Eligible participants were divided into two groups based on their most recent CD4 T-cell levels (within 6 months): those with CD4 T-cell levels >200 cells/mm^3^ and those with CD4 T-cell levels ≤200 cells/mm^3^.

This study was registered in the Thai Clinical Trials Registry (TCTR20240708002) and received approval from the Institutional Review Board of the Faculty of Medicine, Chulalongkorn University (COA No.0813/2023). All of the participants provided written informed consent.

### Study procedures

After providing written informed consent, participants from both groups received a booster dose of the bivalent BA.4/BA.5 vaccine (BNT16b2, mRNA-1273.222). Blood samples were collected before and 4 weeks after the administration of the bivalent vaccine. Serum samples were anonymized, aliquoted, and stored at −30°C until analysis.

We collected data on demographic characteristics (age, gender, Body mass index: BMI), medical comorbidities (chronic heart disease, chronic lung disease, chronic liver disease, chronic renal disease, rheumatologic disease, hypertension, diabetes mellitus, and dyslipidemia), HIV disease and treatment information (years since diagnosis, latest CD4 cell count and percentage, current and previous Antiretroviral (ARV) regimens, history of virologic failure), and COVID-19-related information (vaccination status and details of prior SARS-CoV-2 infections) at baseline.

Adverse reactions to the vaccination were evaluated weekly via follow-up phone calls. Participants who developed COVID-19 infection after enrolment were subsequently excluded from the study.

### Measurement

#### The immunoglobulin G (IgG) anti-receptor binding domain (RBD) antibodies.

Immunoglobulin G (IgG) Anti-receptor binding domain (RBD) of the S1 subunit of the SARS-CoV-2 spike protein were measured using the IgG II Quant assay (Abbott Laboratories, Illinois, USA) according to the manufacturer’s instructions. The assay’s analytical measurement range is 21–40,000 AU/ml, with antibody concentrations ≥50 AU/mL considered positive.

#### The surrogate virus neutralization test (sVNT).

Surrogate Virus Neutralization Test (sVNT) was conducted against the ancestral strain, various Omicron subvariants (BA.1, BA.2, BA.5, XBB.1.5, and XBB.1.16), and other variant of concern (Delta, Delta plus, Lambda, Gamma, Beta, Mu) using the 20-plex sVNT platform [15,16] and the WHO international standard NIBSC code 20/136 [17]. This test evaluates the functionality of antibody by specifically assessing their ability to neutralizes the virus. The procedure involves incubating diluted serum samples (1:320) with receptor binding domain conjugated to horseradish peroxidase (RBD-HRP), which was then transferred to the plate coated with human angiotensin-converting enzyme 2 (hACE2) and incubated at 37°C for 30 minutes. Neutralizing antibodies in the serum competitively inhibit the binding of RBD to hACE2. The remaining HRP-RBD-ACE2 complex is then measured calorimetrically, with absorbance reported as a percentage inhibition relative to a negative control serum sample.

A cut-off value of > 30% signal inhibition indicates seropositivity, as recommended by the manufacturer and validated for detecting neutralizing antibodies against variants of concern [18–20]. Clinical studies have shown excellent concordance between the sVNT and the gold standard 90% plaque reduction neutralization test (PRNT90) for SARS-CoV-2 antibodies detection [21,22].

### Statistical analysis

Power calculations were performed using SAS 9.4 (Cary, NC, USA), based on a coefficient of variation of 4.4 derived from previous study comparing mRNA vaccine responses in PLWH with low (≤200 cells/mm^3^) and high CD4 counts [5]. A total of 116 participants (58 in each CD4 groups) would provide 80% power to detect a reduction in geometric mean antibody level of 60% or more at a 2-sided significance level of 5%.

Statistical analyses were performed by Stata 18 (Statacorp LLC, College Station, TX), with figures generated in Stata and GraphPad Prism v9.4 (IBM Corp, San Diego, CA). Demographic characteristics were presented as frequencies and percentages for categorical variables or as median (interquartile range [IQR]) for continuous variables. Anti-RBD IgG geometric mean titers (GMT) were summarized pre- and post-boost by CD4 group, and the distribution of anti-RBD titers pre- and post-vaccination were graphed by CD4 group. For the primary outcome of post-boost geometric mean titer ratio (GMR) of antibody concentrations, we used a generalized estimating equation (GEE) to analyse the within group change in the natural log- transformed Anti-RBD IgG from pre- to 28 days post boost, with CD4 group as the predictor variable. The model coefficient and 95% confidence interval (95%CI) were exponentiated to derive the GMR and 95%CI.

For secondary outcomes, we described the percentage inhibition of sVNT against XBB, BA.5, Delta and Ancestral strains, with Anti-RBD IgG level, using Spearman’s rank correlation coefficient. We also graphed the distribution of post-vaccination sVNT inhibition by each strain, making formal comparisons by CD4 study group with a Mann-Whitney U test, as well as reporting the percent of seropositive cases.

Finally, we applied a 4-parameter logistic function to model the sigmoidal-shaped concentration-inhibition curve describing the relationship between sVNT inhibition and log10 Anti-RBD IgG levels, using cluster-robust standard errors to account for the within person correlation in pre- and post-boost assay results.

## Results

### Participant characteristics

**Fig 1:**
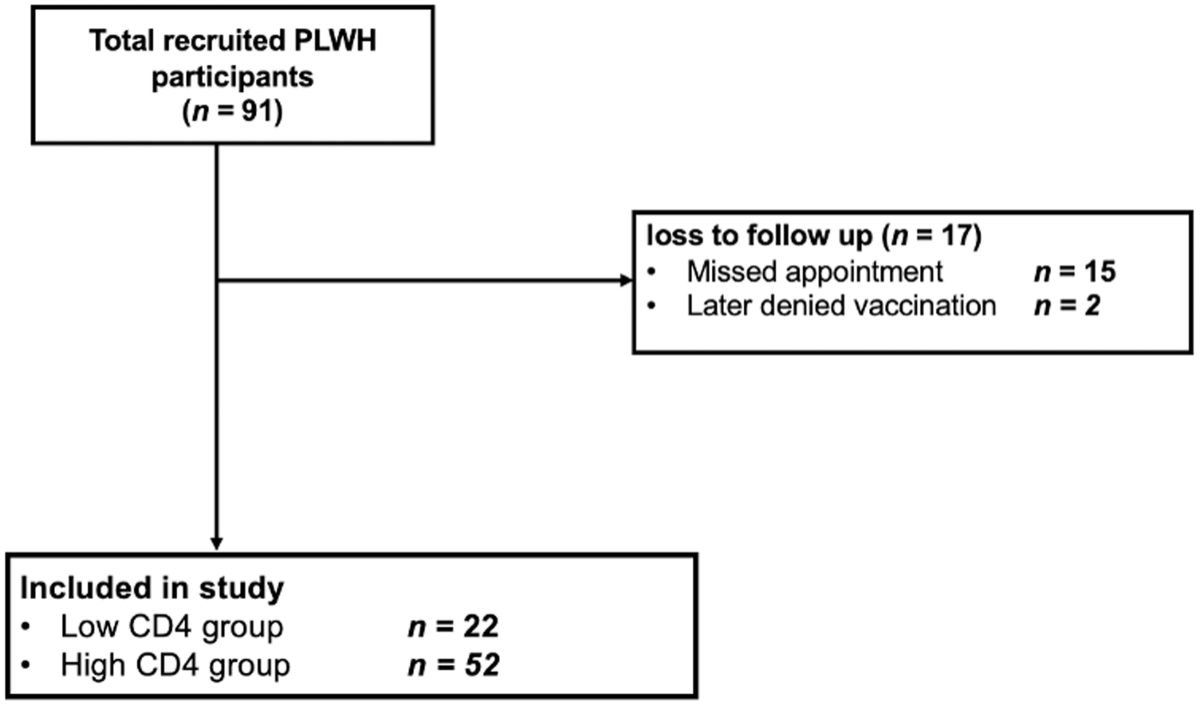
Recruitment flow of study participants for this study.

Baseline characteristics of the study participants are shown in Table 1. Between July 2023 and March 2024, a total of 74 participants were finally recruited (Fig 1), with available immunogenicity data before and four weeks after receiving the bivalent mRNA vaccine booster dose. Of these, 52 participants had CD4 counts > 200 cells/mm^3^ (high CD4 group), and 22 participants had CD4 **≤** 200 cells/mm^3^ (low CD4 group).

**Table 1:** Baseline characteristics of people living with HIV (n = 74) at the time of administration of a bivalent original/omicron BA.4-5 mRNA vaccine booster dose, divided into the low CD4 group (CD4 T-cell levels ≤200 cells/mm^3^) and the high CD4 group (CD4 T-cell levels >200 cells/mm^3^).

Participants characteristic	Low CD4 group (LCD4) CD4 ≤ 200 (N = 22)	High CD4 group (HCD4) CD4 > 200 (N = 52)	P- value
Age, Median (IQR)	54.5 (37.8-61.5)	52 (41.5-59)	0.75
Sex, male (%)	20 (90.9)	32 (65.4)	0.03
BMI (kg/m^2^), Median (IQR)	22.88 (20.74-24.94)	25.59 (22.46-27.63)	0.03
CD4 (cells/mm^3^), Median (IQR)	157 (103- 180)	491 (405- 750)	<0.0001
CD4 (%), Median (IQR)	9.5 (7-12.8)	25 (19- 29.8)	<0.0001
Nadir CD4 * (n), cell/mm^3^, Median (IQR)	(18) 23.5 (14.5- 88)	(40) 87.50 (26.3- 263.3)	0.01
Time since diagnosed HIV (years)	4.5 (1.8- 15)	14.5 (19 -29.8)	0.004
Current ARV regimen, n (%)			
Integrase strand transfer inhibitor (INSTIs) based	18 (81.8)	41 (78.9)	0.78
Protease inhibitors based	1 (4.6)	2 (3.9)	0.89
NNRTI based	3 (13.6)	8 (15.4)	0.84
Other regimen	0	1 (1.92)	0.52
Previous COVID-19 infection, n (%)	11 (50)	30 (57.7)	0.55
Duration from previous infection, n (%)			
3–6 months	1 (0.9)	0 (0)	0.49
6–12 months	1 (0.9)	1 (3.3)	0.55
>12 months	9 (98)	29 (96.7)	0.76
Total previous COVID-19 vaccine doses, n (%)			
2	2 (9.1)	4 (7.7)	0.84
3	14 (63.6)	34 (65.4)	0.88
4	6 (27.3)	14 (26.9)	0.97
Previous mRNA vaccine doses, n (%)			
None	2 (9)	3 (5.7)	0.6
1	11 (50)	37 (71.2)	0.08
2	6 (27.3)	11 (21.2)	0.57
3	3 (13.6)	1 (1.9)	0.04
Duration from last dose (days), Median (IQR)	678 (561–743)	587 (527–670)	0.04
Booster vaccine administered in this study			
Moderna COVID-19 Vaccine, Bivalent (Original and Omicron BA.4/BA.5); mRNA-1273.222, n (%)	0 (0)	14 (26.9)	0.01
Pfizer-BioNTech COVID-19 Vaccine, Bivalent (Original and Omicron BA.4/BA.5); BNT16b2, n (%)	22 (100)	38 (73.1)	0.01

Abbreviations: HIV, human immunodeficiency virus; IQR, interquartile range; BMI, Body mass Index; ARV, Antiretroviral; NNRTI, Non-Nucleoside Reverse Transcriptase Inhibitor

Age distribution was comparable between study groups. The median CD4 count in the high CD4 group was 491 (IQR, 404.5–749.8) cells/mm. Among the 40 participants (76.9%) with available data in this group, the median nadir CD4 cell count was 87.5 (IQR, 26.25–263.3) cells/mm^3^, and median time since HIV diagnosis was 14.5 (IQR, 19–29.8) years. Thirty participants (57.69%) reported a previous COVID-19 infection, with 29 out of 30 (96.7%) reporting that the infection occurred more than 12 months before enrolment. One participant (3.3%) reported previous infection with a duration of 6–12 months before enrolment. Most participants in this group (34 out of 52) had received three total vaccine doses, and 37 (71%) had received at least one previous mRNA vaccine dose. The median duration from enrolment to most recent vaccination was 587 (IQR, 527–670) days.

The median CD4 count in the low CD4 group was significantly lower than the high CD4 cell group at 156.5 (IQR, 103.3–180.0) cells/mm^3^. Among the 18 participants with available data, the median nadir CD4 cell count was 23.5 (IQR, 14.5–88) cells/mm^3^ and median time since HIV diagnosis was 4.5 (IQR, 1.75–15) years. Eleven participants (50%) reported a previous COVID-19 infection; nine of these (98%) reported that the infection occurred more than 12 months before enrolment, while in the remaining two participants, the previous COVID-19 infection occurred 6–12 months and 3–6 months before enrolment, respectively. Most participants in this group (14 out of 22) had previously received three COVID-19 vaccine doses, and 11 (50%) had received an mRNA vaccine. The median duration from enrolment to the most recent vaccination was 678 (IQR, 561–743) days.

### Anti-RBD IgG responses to bivalent booster (Fig 2)

The geometric mean titer (GMT) of Anti-RBD total Ig at baseline (pre-booster) was 687.8 (95% CI, 184.1–2569) AU/mL in the low CD4 group which increased to 13,959 (95% CI, 5,703–34,168) AU/mL post vaccination: a GMR increase of 20.3 (95%CI 7.3–56.2); p<0.001. In the high CD4 cell group, pre-booster titer was 3479 (95%CI 2420–5001) AU/mL which increased to 35,239 (27,363–45,381) AU/mL: a GMR increase of 10.1 (95%CI 7.5–13.7); P<0.001. The post-booster GMR change in the low versus high CD4 group was 2.0 (95%CI 0.9–4.6); P <0.1. Seroconversion rates were 100% in each group, despite a 2.5 lower baseline GMT in the low versus the high CD4 group.

**Fig 2:**
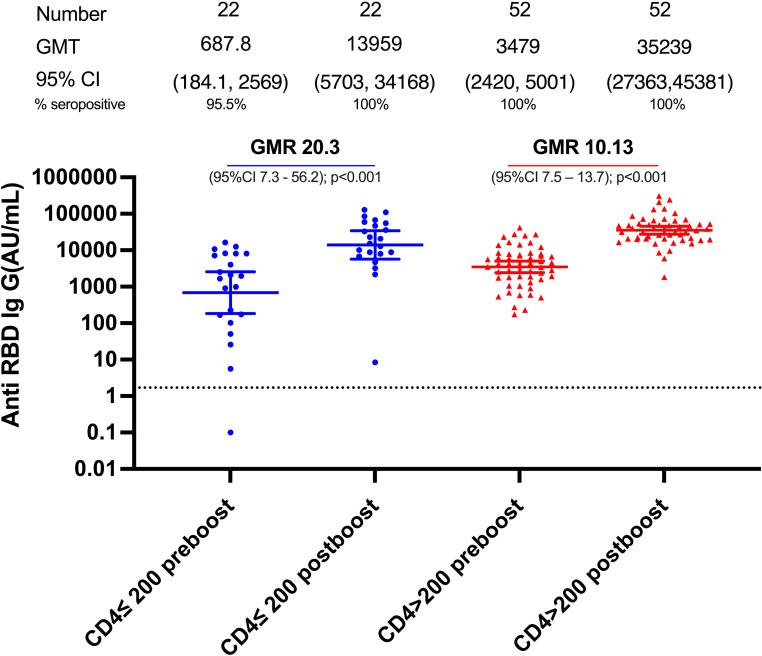
Anti-RBD Ig response after bivalent covid vaccine booster in patients with CD4 ≤ **200 cells/mm**^**3**^ **versus CD4 > 200 cells/mm**^3^. Seropositive rates are based on the manufacturer’s antibody concentrations cut off of ≥50 AU/mL (shown as a dotted line).

### Surrogate virus neutralization test (sVNT) against ancestral, Omicron and other concerning variants

**Fig 3:**
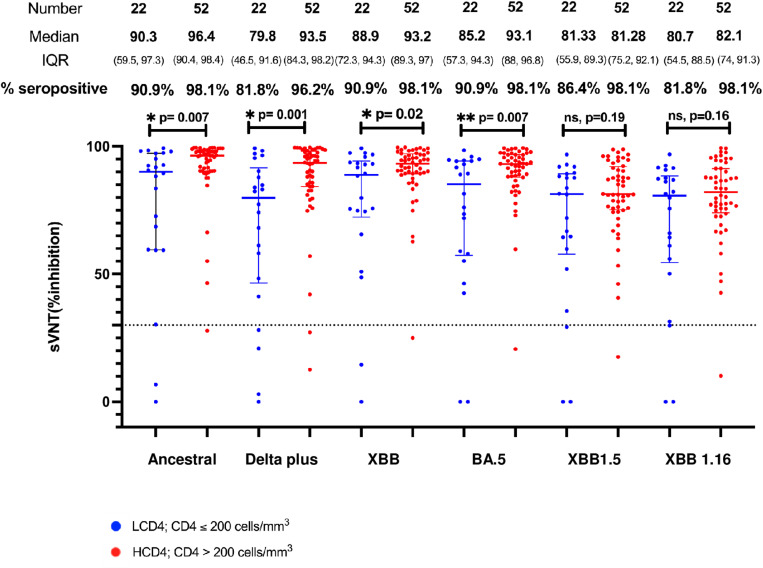
sVNT (% inhibition) of post booster samples for different strains, comparing the low CD4 group (LCD4; CD4 ≤ 200 cells/mm^3^) and the high CD4 group (HCD4; CD4 > 200 cells/mm^3^). Seroconversion rates are based on the manufacturer’s cutoff of 30% sVNT inhibition (shown as a dotted line).

Neutralization against all strains was significantly lower in the low CD4 group compared to the high CD4 group both before and four weeks after the boosting dose. In post-booster samples of both groups (Fig 3), the median % inhibition of sVNT was highest in the ancestral strain (high CD4 group: 96.4% vs. low CD4 group: 90.3%, p-value = 0.007), followed by the Delta plus (high CD4 group: 93.5% vs. low CD4 group: 79.8%, p-value = 0.01), XBB strain (high CD4 group: 93.2% vs. low CD4 group: 88.9%, p-value = 0.02), BA.5 (high CD4 group: 93.1% vs. low CD4 group: 85.2%, p-value = 0.007), XBB 1.5 (high CD4 group: 81.28% vs. low CD4 group: 81.33%, p-value = 0.19), and XBB 1.16 (high CD4 group: 82.1%, low CD4 group: 80.7%, p-value = 0.16).

Dynamic changes of sVNT (% inhibition) against different strains, comparing the high CD4 group and low CD4 group are illustrated in supplementary Fig 1 (S1 Fig).

### Correlation between of anti-RBD IgG and sVNT

Spearman’s rank correlation coefficients were employed to examine the relationship between pre- and post-boost anti-RBD total-IgG (AU/mL) and percentage inhibition of surrogate virus neutralization test (sVNT) against the ancestral strain BA5 (the target strain of the bivalent covid vaccine) and the XBB1.5 and XBB 1.16 strains which were circulating at the time of the study. We found robust correlations between pre-boost anti-RBD Ig levels and sVNT inhibition against these SAR-CoV-2 strains (Spearman’s rho ≥ 0.73). However in all cases, correlation strength reduced post-boost, most markedly for the XBB1.16 variant, while still maintaining significant relationships (S2 Fig).

**Fig 4:**
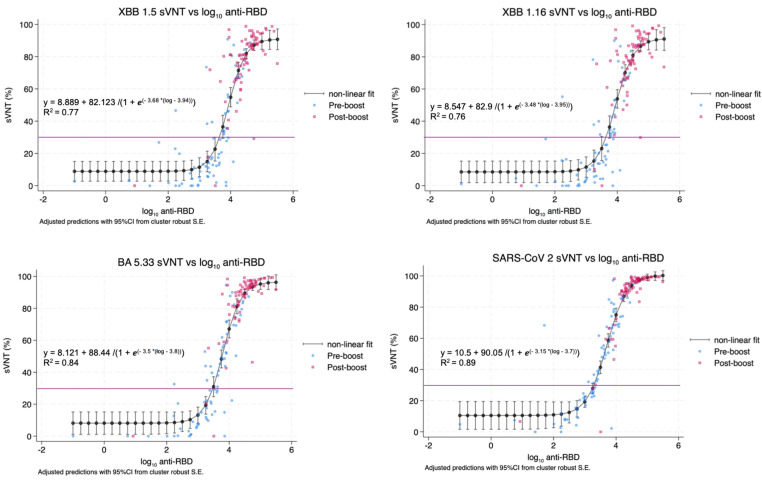
Correlation of predicted sVNT against XBB1.5, XBB1.16, BA.5, Ancestral strain, and log Anti-RBD Ig titers using non-linear regression analysis. The purple lines indicate the manufacturer recommended threshold cutoff of 30%. Graphs are annotated with the non-linear equations for the predicted curves; the R^2^ values show the variability in sVNT percent explained by anti-log_10_ anti-RBD, and vice-versa.

Non-linear regression models were employed to assess the relationship between sVNT percentages and log_10_ anti-RBD IgG titers in both pre and post boost samples, using cluster-robust standard errors. The analysis determined the minimum log_10_Anti-RBD IgG required to achieve 30% of sVNT (the sero-protection cutoff) for different SARS-CoV-2 strains. The required log_10_ anti-RBD IgG titers were 3.3 for the ancestral and 3.49 for the BA.5 strains (vaccine-targeted strains). For XBB1.5, XBB1.16, the required log_10_ anti-RBD IgG was 3.65 (Fig 4).

## Discussion

PLWH are a diverse group, differing in degree of immune deficiency and viral suppression, which impacts their immune response to infection or vaccinations. Current real-world data on COVID-19 vaccine efficacy in PLWH is limited. Similar to other immunocompromised populations including cancer patients, solid organ transplant recipients and those on immunosuppressive therapy, PLWH may have a suboptimal response to COVID 19 vaccine.

Our study sheds light on the antibody response to a bivalent mRNA COVID-19 vaccine booster among PLWH, specifically comparing those with CD4 T-cell levels ≤200 cells/mm^3^ (low CD4 group) to those with higher levels (high CD4 group). Both groups were on effective ART with virologic suppression, and participant in the high CD4 group had been on ART for a longer duration. Importantly, all participants had received their last vaccination over a year prior to the study. The primary COVID19 vaccine in Thailand predominately used inactivated SARS-CoV-2 vaccine (Sinovac-CoronaVac) and ChAdOx1-S vaccine which generally elicited lower antibody response and faster decline compared to mRNA vaccines [23–25]. In mid-2021, mRNA vaccines, such as BNT162b2 and mRNA-1273 were introduced in Thailand, and subsequent mRNA booster recommendations were updated to include variants like BA.4 and BA.5.

With the emergence of the SARS-CoV-2 BA.4 and BA.5 variants, mRNA booster vaccine covering these sublineages were recommended. Our study included participants who received both the original and Omicron BA.4/BA.5 bivalent vaccines (mRNA-1273.222 and BNT162b2). The fact that all participants received the bivalent booster a year after their last vaccination, suggests a low rate of COVID-19 vaccine acceptance and limited awareness of the severity of COVID-19 among PLWH [26]. This low rate of COVID-19 vaccination may lead to a higher hospitalization and mortality rates from severe COVID-19. Our study underscores the need for strategies to promote COVID-19 vaccination in this population.

Our findings revealed that although individuals with low CD4 group had lower baseline Anti-RBD IgG titers compared to those with high CD4 cell counts, both groups showed significant post-boost increases in the GMR. Moreover, the GMR increase in the low CD4 group exhibited a 2-fold higher compared to the high CD4 group. Although this comparison was not statistically significant, the 95% CI around the difference were predominantly consistent with an improved response in the low CD4 group, and influenced by the smaller number of participants in this group [27]. This finding is contrary to previous cohorts [5,6,28], which reported lower GMR in individuals with low CD4 cell counts. This discrepancy may at least partly be attributed to our cohort’s effective virologic suppression, which likely contributed to a more robust immune response despite lower CD4 count. Our study supports that maintaining effective ART plays a crucial role in optimizing vaccine responses in this population. The enhanced antibody response in the low CD4 group might be driven by effective B cell function in virologically suppressed PLWH [29,30]. This contrasts with previous studies where lower CD4 counts were associated with poorer vaccine responses, possibly due to differences in virologic control. After adjusting for the number of previous mRNA doses, the GMR remained consistent across the study population. This finding indicates that the history of prior mRNA vaccination did not significantly impact the primary outcome observed in this study. Given the lower baseline antibody titers and the potential for faster waning of antibody levels in the low CD4 group, additional booster doses and shorter intervals between boosters may help sustain protective immunity. Targeted campaigns to improve vaccine acceptance and personalized strategies incorporating immune monitoring could further optimize protection for PLWH with low CD4 counts. If further studies extend our understanding of antibody longevity, these recommendations could be refined to better address the needs of this population.

In this study, significant sVNT inhibition percentages against various SAR-CoV-2 variants were observed four weeks after the bivalent booster vaccination, suggesting potential cross-immunity from vaccination. Post-booster seropositivity was higher in the high CD4 group for all strains with median sVNT levels significantly higher in the high CD4 group for neutralizing the ancestral, Delta, and BA.5 strains, but not for XBB.1.5 and XBB.1.16. Notably, neutralization levels against XBB.1.5 and XBB.1.16 were lower in both CD4 groups, indicating inadequate cross-neutralization from the bivalent vaccine against these XBB strain. These findings underscore the need to update COVID-19 vaccines to include current circulating SARS-CoV-2 variants for better protection.

A positive correlation between Anti-RBD IgG and sVNT against ancestral, BA.5, XBB 1.16, and XBB 1.5 strains was observed at baseline, likely indicating previous asymptomatic infections or partial cross-immunity. However, this correlation weakened in samples taken four weeks post-vaccination. The moderate response group (GMT of Anti-RBD IgG ranging from 10^4^ to 10^5^ AU/mL) showed increased sVNT inhibition against variants post-boost, which could indicate an improvement in the quality of Anti -RBD IgG, enhancing affinity to SARS-CoV-2 variants. Despite this, some sVNT responses remained low, suggesting diverse immunogenicity but not necessarily strain-specific responses. The variability in correlation, particularly with XBB 1.5 and 1.16, suggests imperfect cross-reactivity from the vaccine.

Non-linear regression models predicted higher seroprotection (sVNT inhibition >30%) against vaccinated strain (ancestral, BA.5) at lower log Anti-RBD IgG titers compared to Omicron circulating strain. These results highlight the difficulty of achieving broad immunity against diverse SARS-CoV-2 variants with a single vaccine formulation.

Previous studies have shown that PLWH with higher CD4 counts and CD4/CD8 ratios, who effectively controlled their HIV viral load and received mRNA COVID-19 vaccines, exhibit better immune responses [31,32]. However, these studies had limitations, including small sample sizes of patients with CD4 counts below 200 cells/mm^3^, variations in the timing of immune globulin measurement, and vaccination type. Additionally, specific neutralizing antibody measurements using standardized pseudovirus neutralization assays (sVNT) [33] or SARS-CoV-2 Spike(S)-Pseudotyped Virus Neutralization Assay (PsVNA) [34,35], and T cell responses, were not measured [31,32].

Our study demonstrates the robust immunogenicity of the bivalent COVID-19 vaccine in virologic suppressed PLWH, with a superior response in those with higher CD4 levels. Despite lower CD4 levels, the low CD4 group achieved a high GMR increase, contrasting with previous studies where lower CD4 levels were not found to influence response [28]. These findings strongly support mRNA COVID-19 vaccination in PLWH with low CD4 counts, though T cell function, which also contributes to protection against severe SARS-CoV-2 infection, was not explored. Data on the durability of antibody responses after bivalent mRNA COVID-19 boosters in PWH, particularly those with advanced HIV, remains limited, with waning neutralizing antibodies observed at 5–6 months[36–38]. Longer protective antibody responses in the low CD4 group with virologic suppression require further confirmation at the 6-month. The bivalent vaccine boosters in this study included both Original and Omicron BA.4/BA.5 (BNT162b2) and Original and Omicron BA.4/BA.5 (mRNA-1273.222). Even after adjusting for vaccine type, a higher response was still observed in the high CD4 group. This cross-sectional study suggests further investigation into the durability of Anti-RBD IgG and sVNT levels across different CD4 groups, vaccination heterogeneity, and predicting sVNT from Anti-RBD IgG titers to better understand booster necessity and timing of booster.

Strengths of our study include the prospective design with no losses to follow-up, and an assessment of anti-RBD titers and sVNT to a range of SARS CoV 2 variants in patients with low and high CD4 counts. Nevertheless, our study has some limitations. First, as noted in the previous paragraph, durability of response could not be determined from our study. Second, as an observational study, our results are subject to unobserved confounding. Third, despite our best efforts we were unable to recruit sufficient eligible participants over the study to satisfy the numbers mandated by the power calculations. However, our sample size was calculated based on antibody responses to a booster dose of the primary mRNA vaccine series [5], whereas most participants in our study had received 3 or more doses and this may account for the high post-vaccination titers observed.

In conclusion, our results support the existing recommendation for mRNA vaccine boosters in PLWH. Although individuals in the low CD4 group exhibited reduced protection at baseline, they showed significant increase in Anti RBD IgG and sVNT levels across all CD4 counts after receiving the bivalent COVID vaccine booster. However, the limited cross-immunity against the XBB strain suggests a need for updated vaccine that target current variants.

## Supporting information

S1 Data Raw data of participants. (XLSX)

S1 Fig Dynamic sVNT (% inhibition) changes against different strains, in the high CD4 group (HCD4) and low CD4 group (LCD4). (TIF)

S2 Fig The correlation between anti-RBD total Ig and sVNT against vaccinated strains: ancestral strain, BA.5 and circulating strains: XBB1.5, XBB1.16. (TIF)

S1 File Research proposal (English translation). (DOCX)

S2 File Research proposal (Thai). (DOCX)

S1 Checklist TREND statment checklist. (PDF)
