# Supplementary figures and images for: Differential immunogenicity in people living with HIV with varying CD4 levels after bivalent mRNA COVID-19 booster vaccination
Source: PLoS One. 2025 Apr 29;20(4):e0317940. doi: 10.1371/journal.pone.0317940 (PMC12040274; doi:10.1371/journal.pone.0317940)

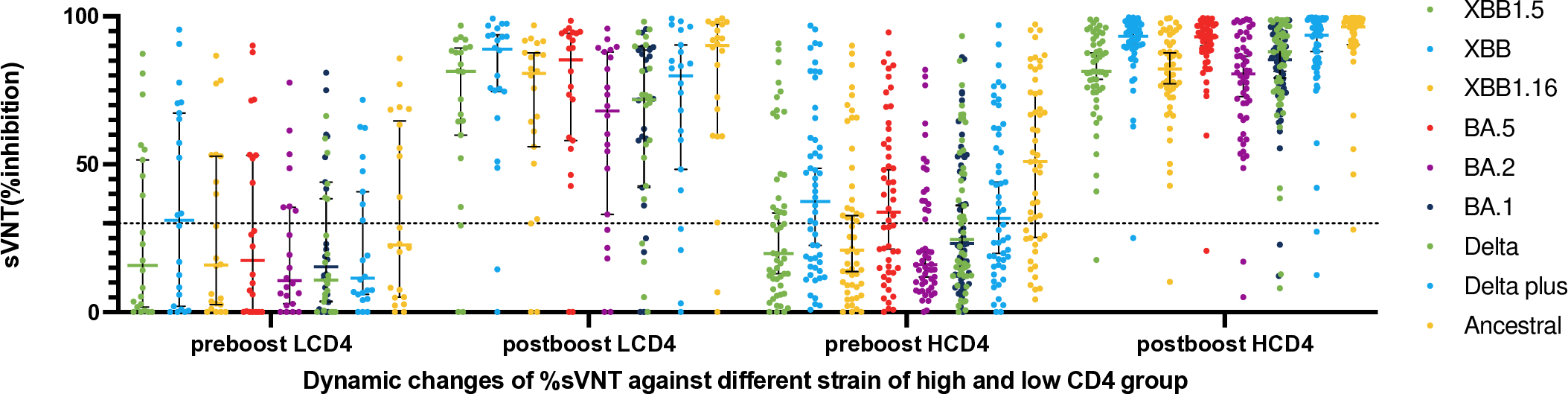

Supplement: S1 Fig — (TIF) [file pone.0317940.s002.tif]

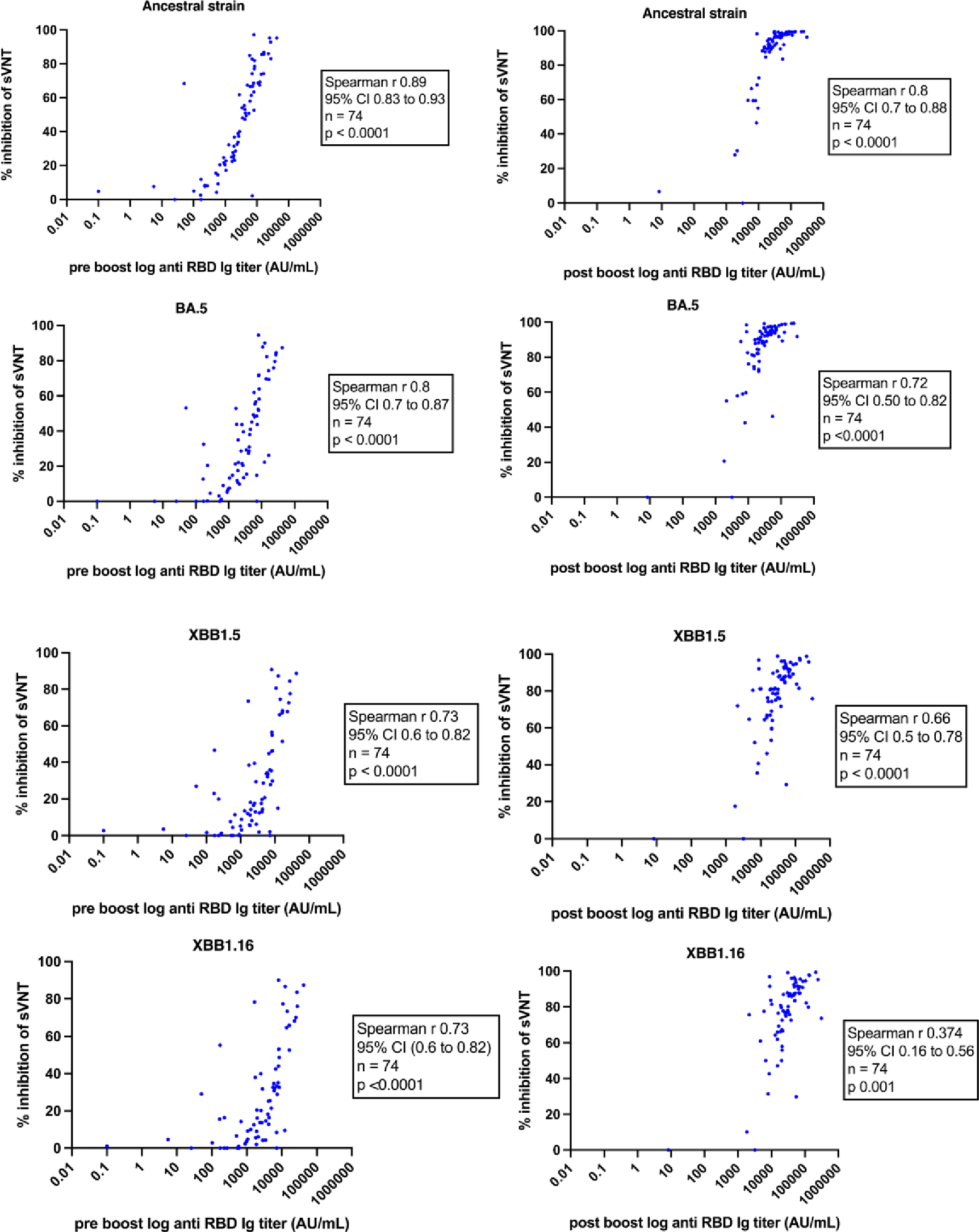

Supplement: S2 Fig — (TIF) [file pone.0317940.s003.tif]
