# Supplementary material for: Differential immunogenicity in people living with HIV with varying CD4 levels after bivalent mRNA COVID-19 booster vaccination
Source: PLoS One. 2025 Apr 29;20(4):e0317940. doi: 10.1371/journal.pone.0317940 (PMC12040274; doi:10.1371/journal.pone.0317940)
Supplement: S1 File — (DOCX) [file pone.0317940.s004.docx]

**Research proposal:
Antibody Response in People living with HIV: Comparing CD4 T-cell Levels >200 vs. ≤200 cells/mm^3^ after mRNA COVID-19 Vaccine Booster**

Napon Hiranburana, M.D.^1^; Opass Putcharoen, M.D. , M.Sc.^1,2^

^1^ Division of Infectious Diseases, Department of Medicine, King Chulalongkorn Memorial Hospital, Thai Red Cross Society, Bangkok, Thailand.

^2^ Thai Red Cross Emerging Infectious Diseases Clinical Center, King Chulalongkorn Memorial Hospital, Bangkok, Thailand

**Background and Rationale**

Due to HIV infection being an independent risk factor for increasing the severity of COVID-19 and increasing the mortality rate of patients^1,2,6^. Preventive measures at the primary level or COVID-19 vaccination have been evidenced to help reduce the severity of COVID-19 when infected.

The efficacy of the COVID-19 vaccine primary series was assessed through the study of immune response (Anti RBD IgG) in HIV-infected individuals after receiving the COVID-19 vaccine. It was found that the immune response was lower compared to the general population and comparable to other groups with low immunity, such as patients with autoimmune diseases and cancer receiving chemotherapy. However, the response was better than those who had organ or bone marrow transplants. Additionally, the post-vaccination Anti RBD IgG levels were moderate^3-5^, and in detail, the ability of antibodies to neutralize (neutralization level) was lower in affinity compared to the group with high levels of Anti RBD IgG. This implies that HIV-infected individuals in the study generally had a moderate level of antibody response.^3,4,8^

When looking at HIV-infected individuals themselves, it was found that the immune response data after receiving the mRNA COVID-19 vaccine primary series was correlated with the number of CD4 T lymphocytes, which may indicate the status of virus control with antiretroviral therapy, and the immunity of the patients. The group with higher CD4 counts had higher overall levels of Anti RBD IgG and better neutralization ability.^4^

There has been development of a bivalent vaccine consisting of mRNA from both the original strain and BA.4, BA.5 strain that has undergone Phase 2 studies.^12^ In the data from Qian Wang et al.,^15^ a study on the immune response after administering the bivalent COVID vaccine was conducted regarding its ability to generate neutralizing antibodies (nAb) specific to various strains, including BA.4–BA.5, BA.4.6, BA.2.75, and BA.2.75.2, which were circulating at that time. It was found that there was no statistically significant difference between the group receiving mRNA vaccine (monovalent) as the fourth dose and the group receiving the bivalent booster as the fourth dose. However, the study population consisted of only 20 individuals per group, and it did not include individuals with compromised immunity.

Therefore, this study aimed to gather information on the immune response after bivalent mRNA COVID-19 vaccine booster shots in HIV-infected individuals with CD4 T lymphocyte counts below or equal 200 cells/mm^3^ compared to those with counts above 200 cells/mm^3^. The study measured responses in terms of overall immunoglobulin levels (Anti RBD IgG) and SARS-CoV-2 surrogate virus neutralization test (sVNT) against new variants of COVID-19 currently circulating, such as the XBB, BA.5, and BA.2 variants. The study also aimed to determine the relationship between overall immunoglobulin levels and neutralization, as well as to identify other factors related to the immune response to the vaccine in HIV-infected individuals.

**Research Questions**

**Primary Research Question:**

Will patients with CD4 counts of 200 cells/mm³ or lower have a lower antibody response, in terms of total immunoglobulin (Anti-RBD total Ig levels), 4 weeks after receiving a booster dose of mRNA vaccine, compared to those with CD4 counts greater than 200 cells/mm³?

**Secondary Research Questions:**

Will patients with CD4 counts of 200 cells/mm³ or fewer have a lower immune response, as measured by neutralizing antibodies (nAb) through the percentage of inhibition in sVNT (surrogate Virus Neutralization Test) against new COVID-19 variants such as XBB, BA.2.75, and the original strain, 4 weeks after receiving the second booster dose of mRNA vaccine, compared to those with CD4 counts greater than 200 cells/mm³?

Will the relationship between total immunoglobulin (Anti-RBD total Ig levels) 4 weeks after receiving a booster dose of mRNA vaccine and the percentage of inhibition in sVNT (surrogate Virus Neutralization Test) in patients with CD4 counts of 200 cells/mm³ or fewer be weaker compared to those with CD4 counts greater than 200 cells/mm³?

Will the increase in total immunoglobulin (Anti-RBD total Ig levels) 4 weeks after receiving a booster dose of mRNA vaccine, compared to before the booster dose, be lower in patients with CD4 counts of 200 cells/mm³ or fewer compared to those with CD4 counts greater than 200 cells/mm³?

**Research Objectives**

**Primary Objective:**

To evaluate the immune response in terms of total immunoglobulin (Anti-RBD total Ig levels) 4 weeks after receiving a booster dose of mRNA vaccine in patients with CD4 counts of 200 cells/mm³ or fewer, compared to those with CD4 counts greater than 200 cells/mm³.

**Secondary Objectives:**

To study the immune response by measuring neutralizing antibodies (nAb) against new COVID-19 variants such as XBB, BA.2.75, 4 weeks after receiving a booster dose of mRNA vaccine, comparing patients with CD4 counts of 200 cells/mm³ or fewer to those with CD4 counts greater than 200 cells/mm³. This will be done using ELISA-based standardized pseudovirus neutralization assays, also known as surrogate virus neutralization tests (sVNT), to measure the relationship between the percentage of inhibition in sVNT and total immunoglobulin (Anti-RBD total Ig levels), as well as the increase in total immunoglobulin (Anti-RBD total Ig levels) 4 weeks after receiving a booster dose of mRNA vaccine compared to before the booster dose.

**Hypothesis:**

Patients with CD4 counts of 200 cells/mm³ or fewer will have a lower immune response in terms of total immunoglobulin (Anti-RBD total Ig levels) after receiving a booster dose of mRNA vaccine, compared to those with CD4 counts greater than 200 cells/mm³. The comparison will be made using a geometric mean ratio (GMR), which is expected to be less than 0.4.

**Conceptual Framework**

**
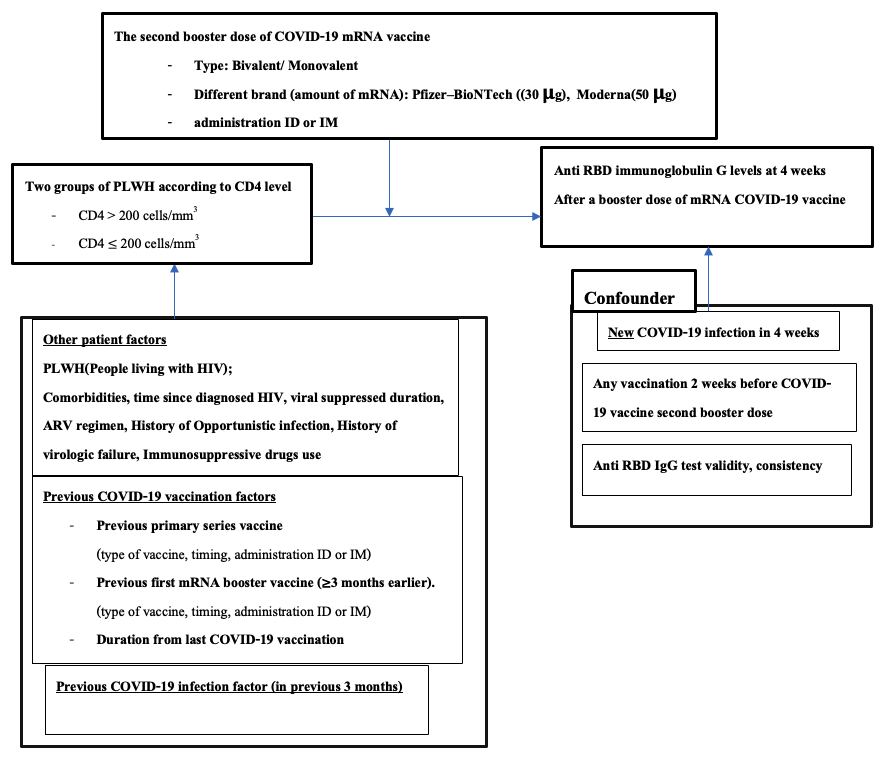
**

**Key words**

Immunogenicity; Antibody response; SARS-CoV-2 ; Anti–SARS-CoV-2 vaccine; HIV/AIDs; CD4 level; Surrogate virus neutralization test

**Operational Definitions to be Used in the Research**

- Participants suspected of having had a COVID-19 infection within the past 3 months: Defined as participants who have had respiratory symptoms consistent with a viral infection and a confirmed positive result from an ATK (Antigen Test Kit) test (whether conducted at a healthcare facility or elsewhere) or a nasal/nasopharyngeal swab PCR test for SARS-CoV-2 within 3 months before the date of participation in the study.
- HIV-positive individuals with suppressed viral load: Defined as HIV-positive individuals with an undetectable HIV viral load (<20 copies/ml) within 1 year.
- HIV-positive individuals with virologic failure: Defined as those with an HIV viral load greater than 200 copies/ml.
- HIV-positive individuals on continuous antiretroviral therapy: Defined as those who have been on monitored antiretroviral therapy for more than 6 months.

**Research Design**: Prospective observational cohort study

**Research Methodology**

**Population:** HIV-positive individuals in the outpatient clinic who are over 18 years old and eligible for a booster dose of the mRNA COVID-19 vaccine.

**Target Population**: HIV-positive individuals in the outpatient clinic who are over 18 years old, on continuous antiretroviral therapy, without virologic failure, and with a CD4 count of 200 cells/mm³ or fewer.

**Control Population:** HIV-positive individuals in the outpatient clinic who are on continuous antiretroviral therapy, without virologic failure, and with a CD4 count greater than 200 cells/mm³.

**Approach to Participants:** The approach will be to contact HIV-positive individuals who come for check-ups at the Infectious Disease Clinic, Pranya Laksana Pukk Building, 14th Floor, King Chulalongkorn Memorial Hospital, and at HIV-NAT (The HIV Netherlands Australia Thailand Research Collaboration) under the Thai Red Cross AIDS Research Centre. Participants will be recruited through posted announcements and telephone inquiries to gauge patient interest.

**Inclusion criteria**

1. PLWH in the outpatient clinic who are over 18 years old and have a suppressed viral load.
2. Participants have been on continuous and the same antiretroviral therapy for at least 3 months.
3. Participants have previously received a COVID-19 vaccine, with at least a primary series of any type (minimum of 2 doses), with the last dose being administered at least 3 months before joining the study.

**Exclusion criteria**

1. Participants had a COVID-19 infection within 3 months prior to joining the study (screened by history and ATK results).
2. Participants have received any other vaccine within 15 days prior.
3. Participants have contraindications to vaccination or a history of allergic reaction to a previous COVID-19 vaccine.
4. Participants are in a state of high-level immunosuppression, such as high-dose immunosuppressive therapy (e.g., prednisolone > 20 mg/day, rituximab, TNF alpha blockers, MTX > 0.4 mg/kg/week).
5. Participants have received monoclonal antibodies, JAK inhibitors, or convalescent plasma for COVID-19.
6. Participants have an active autoimmune disease or are currently undergoing treatment for any type of cancer.

**Sample size calculation**

Power calculations are based on a number of assumptions: we assume the post-boost antibody titres will be lognormally distributed, with a coefficient of variation (CV) of 4.4 (or geometric standard deviation of 1.71).  This CV is derived from a recent publication in CID ^4^ comparing primary vaccine responses people with low (≤200 cells/mm 3 ) and high CD4 counts.  It is also consistent with the CV for overall antibody increases after a booster dose of vaccine in a cohort of Chulalongkorn Oncology Clinic patients who were treated with a variety of different agents.^13^  We would like 80% power to detect a 60% reduction in geometric mean antibody

concentration in the low CD4 versus the high CD4 count group as a reference.

Sample size for a 2 group comparison of means with equal allocation ratio can be

derived with the following formula:

$$\frac{\left( Z_{1-\alpha/2}+Z_{1-\beta} \right)^{2}\left( \sigma^{2} \right)}{\Delta^{2}}$$

Where $Z_{1-\alpha/2}$ is the critical value for significance and equals 1.96 at a 5% significance level

$Z_{1-\beta}$ is the critical value for power and equals 0.842 at 80% power, $\sigma$ is the geometric standard deviation, and $\Delta$ is the natural log transformed geometric mean ratio for the low CD4 group versus the high CD4 group as a reference (log(0.4) = 0.91).

A total of 116 patients (58 each in the high and low CD4 groups) would 80%

power to detect a reduction in geometric mean antibody of 60% or greater, at a 2-

sided significance level of 5%. SAS output for this calculation is shown below.

Given the very short period between boosting dose and outcome assessment, we

believe losses to follow-up will be minimal, so sample sizes have been inflated by

5% to a total of 122 (61 per group) patients.


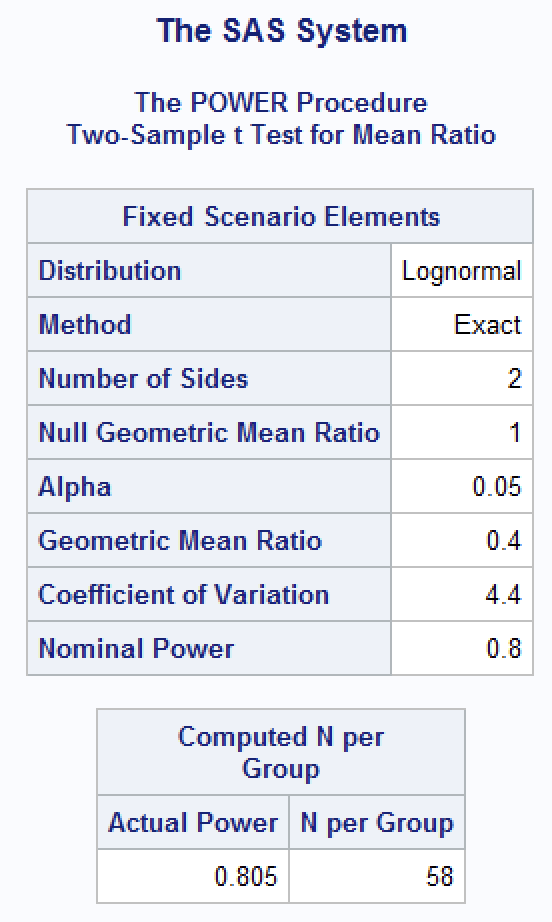


**Informed Consent Process**

The informed consent process will take place at King Chulalongkorn Memorial Hospital and HIV-NAT. The research physician will explain the research procedures, risks, and benefits, and answer any questions until the volunteer fully understands. The volunteer will then be given time to make an independent decision before signing the consent form to participate in the research.

**Research Methodology**

**Data Collection**

1. The research team will collect data from HIV-positive individuals eligible for a second booster dose of mRNA COVID-19 vaccine at the Infectious Disease Outpatient Clinic, 14th Floor, Pranya Laksana Pukk Building, and HIV-NAT
2. Assess whether the patients meet the inclusion and exclusion criteria.
3. The research team will explain the research procedures to the volunteers as follows
4. The volunteers will read and sign the informed consent form, with the date indicated.
5. The research team will collect the following data:
   - - Personal information: Gender, age
     - Other underlying diseases
     - The latest CD4 count within 6 months at the clinic and the lowest level, as well as the CD4/CD8 ratio (if available)
     - Duration since HIV diagnosis
     - Antiretroviral therapy regimen and duration on antiretroviral therapy
     - History of opportunistic infections and virologic failure after starting treatment, based on available data
     - History of prior COVID-19 vaccinations
     - History of other vaccinations
6. Schedule the first blood sample collection (15 ml) to test for:
   - - CD4, CBC
     - Anti RBD total Ig
     - sVNT (% inhibition level) against new COVID-19 variants such as XBB, BA.2.75, and the original strain
7. Administer the bivalent mRNA COVID-19 booster vaccine on the same day after the first blood sample collection and monitor for side effects at the vaccination site for 30 minutes.
8. Schedule the second blood sample collection (15 ml) 28 days after vaccination (Anti RBD total Ig and sVNT (% inhibition level))
9. During the waiting period for the second blood test, the research team will follow up with the patients weekly by phone to check for symptoms suggestive of COVID-19 infection and potential vaccine side effects.
10. For patients with suspected COVID-19 infection and positive ATK results, if they are at risk for severe disease and meet the criteria for COVID-19 antiviral therapy, they will be contacted to receive services at King Chulalongkorn Memorial Hospital.Such patients will be excluded from the study.
11. Patients without suspected COVID-19 symptoms will be asked to come for the second blood test as scheduled.
12. Data analysis and research report preparation.


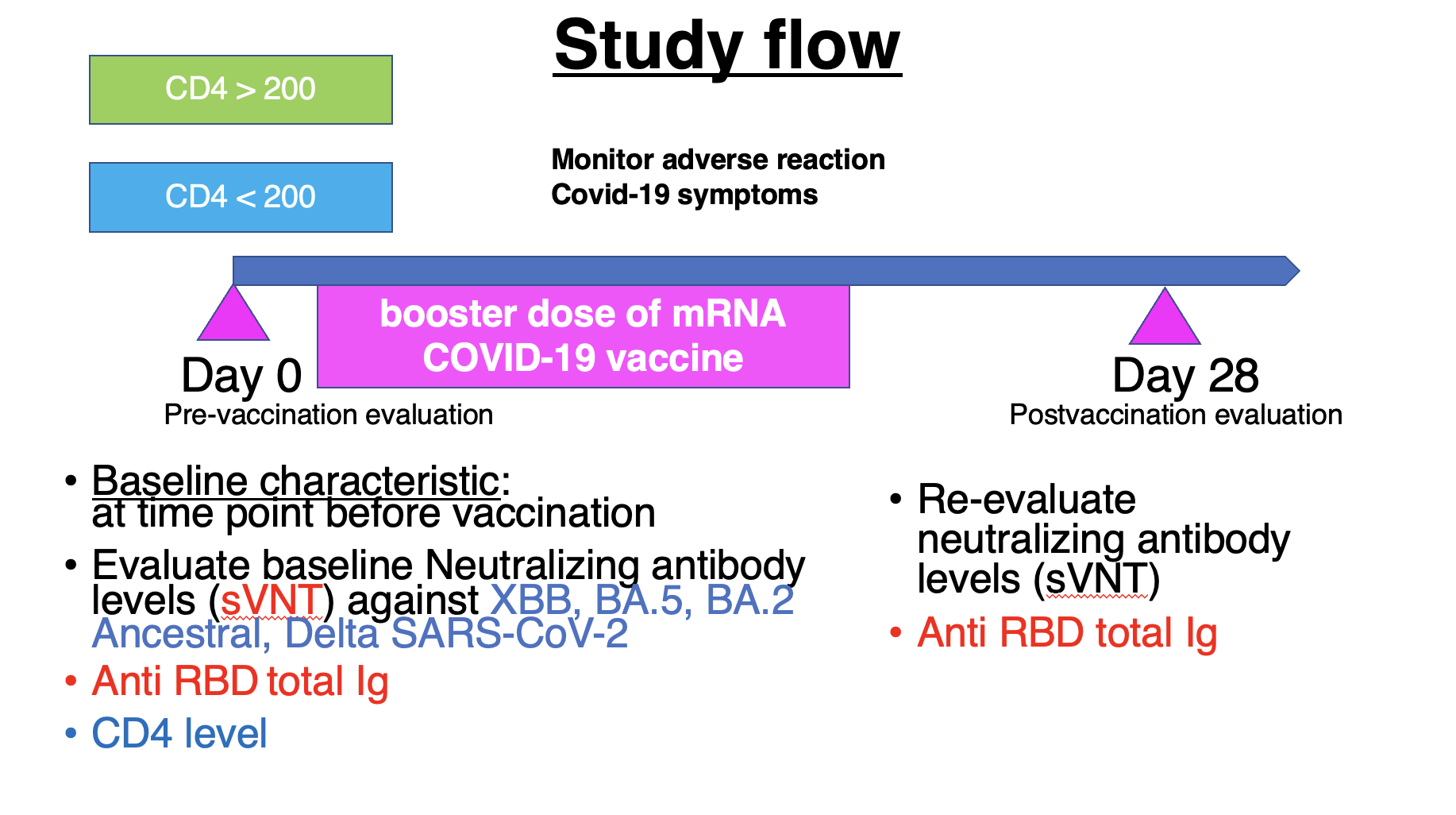


**Measurement**

We measured immunoglobulin G (IgG) anti-receptor binding domain (RBD) antibodies and surrogate Virus Neutralization Test (sVNT) against WT and Omicron subvariants (BA.1, BA.2, BA.5, XBB.1.5, and XBB.1.16) before and after the bivalent booster.

**The immunoglobulin G (IgG) anti-receptor binding domain (RBD) antibodies**

The SARS-CoV-2 IgG II Quant assay is an automated, two-step chemiluminescent microparticle immunoassay (CMIA) from Abbot company. It is used for the qualitative and quantitative determination of IgG antibodies to the receptor binding domain (RBD) of the S1 subunit of the spike protein of SARS-CoV-2 in human serum and plasma on the Alinity i system. The sequence used for the RBD was taken from the WH-Human 1 coronavirus, GenBank accession number MN908947. The analytical measurement interval is stated as 21 to 40,000 AU/ml, and positivity cutoff is ≥50 AU/ml (manufacturer defined).

**The surrogate Virus Neutralization Test (sVNT)**

The cPass^TM^ sVNT is evaluated by ELISA technique from GenScript company. It is used to evaluate the function of antibody that specifically neutralizes the virus. Briefly, patient samples were mixed with the receptor binding domain conjugated to horseradish peroxidase (RBD-HRP) and then transferred to the plate coated with human angiotensin-converting enzyme 2 (hACE2) before incubation at 37^o^c for 30 minutes.

A low OD_450_ will be read after adding 3,3′,5,5′-tetramethylbenzidine (TMB) and incubating for 15 minutes, followed by the addition of the stop solution. The serum with neutralizing antibodies will block the RBD - hACE2 interaction and will be washed out from the plate, giving a color ranging from colorless to light-yellow color after adding the stop solution. On the other hand, if the serum does not contain neutralizing antibodies, RBD will bind to the hACE2 and show a yellow color after the addition of the stop solution.

The sVNT is reported in percent signal inhibition that is calculated from the equation below.

% inhibition = $1-\frac{\mathrm{OD} \mathrm{sample}}{\mathrm{OD} \mathrm{negative} \mathrm{control}}x 100\%$

A result below 30% signal inhibition indicates seronegativity to the virus causing COVID-19.^16^

**Analysis plan**

For the primary outcome of geometric mean ratio antibody concentrations, we will use a regression model with outcome of natural log transformed anti-RBD IgG titres 28 days after boosting, and predictor variable of CD4 group. The model coefficient and 95%CI will be exponentiated to derive the geometric mean ratio (GMR) and 95%CI. If there are important differences in the group demographic or disease-related characteristics, we will also develop adjusted models.

For the secondary outcomes, we will describe the percentage inhibition of sVNT against XBB, BA.5, delta and wild type virus, and formal comparisons of the percentage inhibition will be made using an independent proportions test. We will assess the correlation of sVNT with total anti-RBD IgG overall, and by CD4 group.

Finally, we will use generalised linear models to describe the relationship between

increasing CD4 cell count as a continuous variable, total anti-RBD IgG, and sVNT percent

inhibition, after adjustment for important confounders.

**Ethics**

This study received approval from the Institute Review Board of the Faculty of Medicine, Chulalongkorn University (IRB No. 0292/66).

**Administration & Time Schedule**

| Activity | 2022 | | | | | | 2023 | | | | | | | | | | | | | | | | | | 2024 | | | | | | | | | | |  |
| --- | --- | --- | --- | --- | --- | --- | --- | --- | --- | --- | --- | --- | --- | --- | --- | --- | --- | --- | --- | --- | --- | --- | --- | --- | --- | --- | --- | --- | --- | --- | --- | --- | --- | --- | --- | --- |
|  | 7 | 8 | 9 | 10 | 11 | 12 | 1 | 2 | 3 | 4 | 5 | | 6 | 7 | | | 8 | 9 | | | 10 | 11 | | 12 | 1 | | 2 | 3 | | 4 | | 5 | | | 6 | |
| Study preparation | x | x | x | x | x | x | x | x | x | x | x | x | | |  |  | | |  |  | | |  |  |  |  | | |  | |  | |  |  | |  |
| Data collection |  |  |  |  |  |  |  |  |  |  |  |  | | | x | x | | | x | x | | | x |  |  |  | | |  | |  | |  |  | |  |
| Data analysis |  |  |  |  |  |  |  |  |  |  |  |  | | |  |  | | |  |  | | |  | x | x |  | | |  | |  | |  |  | |  |
| Research report |  |  |  |  |  |  |  |  |  |  |  |  | | |  |  | | |  |  | | |  |  |  | x | | | x | | x | | x | x | |  |

**Budget**

It is expected that all booster doses of the mRNA vaccine will be provided by the government, and the budget for immunological response testing and compensation for volunteers will come from the Thai AIDS society.

| **Expense Category** | **Budget (THB)** |
| --- | --- |
| 1. Personnel Budget | 0 |
| 2. Operating Budget |  |
| - Data collection and analysis | 10,000 |
| - Travel expenses for 122 COVID-19 patient volunteers, 300 THB each for 1 time | 36,600 |
| - Cost of Anti RBD total Ig test, 500 THB per sample, total of 152 samples | 122,000 |
| **Total (THB)** | 168,600 |

**Appendix: Dummy Table**

| **Characteristic** | **CD4 < 200 cells/mm³** | **CD4 > 200 cells/mm³** | **p-value** |
| --- | --- | --- | --- |
| Gender |  |  |  |
| Age, median |  |  |  |
| Current CD4 count, cells/mm³, median |  |  |  |
| Time since HIV diagnosis (years), median |  |  |  |
| History of virologic failure (%) |  |  |  |
| Antiretroviral regimen (RTIs, PIs, INSTIs, %) |  |  |  |
| History of prior COVID-19 vaccination |  |  |  |
| Number of mRNA vaccine doses, median |  |  |  |

References

1. Bertagnolio S, Thwin SS, Silva R, et al. Clinical features of, and risk factors for, severe or fatal COVID-19 among people living with HIV admitted to hospital: analysis of data from the WHO Global Clinical Platform of COVID-19. Lancet HIV. In press.
2. TesorieroJM,SwainCE,PierceJL,etal.COVID-19 outcomes among persons living with or without diagnosed HIV infection in New York state. JAMA Netw Open 2021; 4:e2037069.
3. Haidar G, Agha M, Bilderback A, et al. Prospective evaluation of COVID-19 vaccine responses across a broad spectrum of immunocompromising conditions: the COVICS study. Clin Infect Dis 2022; 75:e630–44.
4. AntinoriA,CicaliniS,MeschiS,etal;HIV-VAC StudyGroup.Humoral and cellular immune response elicited by mRNA vaccination against SARS-CoV-2 in people living with HIV (PLWH) receiving antiretroviral therapy (ART) according with current CD4 T-lymphocyte count. Clin Infect Dis 2022; 75:e552–63.
5. Hassold N, Brichler S, Ouedraogo E, et al. Impaired antibody response to
   COVID-19 vaccination in advanced HIV infection. AIDS 2022; 36:F1–5.
6. Bhaskaran K, Rentsch CT, MacKenna B, et al. HIV infection and COVID-19 death: a population-based cohort analysis of UK primary care data and linked national death registrations within the OpenSAFELY platform. Lancet HIV 2021; 8:e24–32.
7. FengY,ZhangY,HeZ,etal.Immunogenicity of an inactivatedSARS-CoV-2vaccine in people living with HIV-1: a non-randomized cohort study. EClinicalMedicine 2022; 43:101226.
8. Xu X, Vesterbacka J, Aleman S, Nowak P; COVAXID Study Group. High sero- conversion rate after vaccination with mRNA BNT162b2 vaccine against SARS-CoV-2 among people with HIV—but HIV viremia matters? AIDS 2022; 36:479–81.
9. Luo YR, Yun C, Chakraborty I, Wu AHB, Lynch KL. A SARS-CoV-2 Label-Free Surrogate Virus Neutralization Test and a Longitudinal Study of Antibody Characteristics in COVID-19 Patients. *J Clin Microbiol*. 2021;59(7):e0019321.
10. Nie J, Li Q, Wu J, et al. Establishment and validation of a pseudovirus neutralization assay for SARS‐CoV‐2. Emerg Microbes Infect. 2020;9(1):680‐686.
11. Focosi D, Maggi F, Mazzetti P, Pistello M. Viral infection neutralization tests: A focus on severe acute respiratory syndrome-coronavirus-2 with implications for convalescent plasma therapy. *Rev Med Virol*. 2021;31(2):e2170.
12. Chalkias S, Harper C, Vrbicky K, et al. A Bivalent Omicron-Containing Booster Vaccine against Covid-19. N Engl J Med. 2022;387(14):1279-1291.
13. Teeyapun N, Luangdilok S, Pakvisal N, et al. Immunogenicity of ChAdOx1-nCoV-19 vaccine in solid malignancy patients by treatment regimen versus healthy controls: A prospective, multicenter observational study. EClinicalMedicine. 2022;52:101608.
14. Chun HM, Milligan K, Agyemang E, et al. A Systematic Review of COVID-19 Vaccine Antibody Responses in People With HIV. Open Forum Infect Dis. 2022;9(11):ofac579.
15. Wang Q, Bowen A, Valdez R, et al. Antibody Response to Omicron BA.4-BA.5 Bivalent Booster [published online ahead of print, 2023 Jan 11]. *N Engl J Med*. 2023
16. Padoan A., Bonfante F., Pagliari M., Bortolami A., Negrini D., Zuin S., Bozzato D., Cosma C., Sciacovelli L., Plebani M. Analytical and clinical performances of five immunoassays for the detection of SARS-CoV-2 antibodies in comparison with neutralization activity. EBioMedicine. 2020;62:103101. doi: 10.1016/j.ebiom.2020.103101.
