# Supplementary material for: Differential immunogenicity in people living with HIV with varying CD4 levels after bivalent mRNA COVID-19 booster vaccination
Source: PLoS One. 2025 Apr 29;20(4):e0317940. doi: 10.1371/journal.pone.0317940 (PMC12040274; doi:10.1371/journal.pone.0317940)
Supplement: S2 File — (DOCX) [file pone.0317940.s005.docx]

**โครงร่างการวิจัย (Research Proposal)**

1. **ชื่อโครงการ (Proposal Title)**

(ภาษาไทย) การตอบสนองของแอนติบอดีหลังวัคซีนโควิด-19 ชนิดเอ็มอาร์เอ็นเอ เข็มกระตุ้นในผู้อยู่ร่วมกับเชื้อเอชไอวี เปรียบเทียบระหว่างกลุ่มที่ระดับเม็ดเลือดขาวชนิดCD4 มากกว่า 200 เซลล์/มม.^3^ และ น้อยว่าหรือเท่ากับ200 เซลล์/มม.^3^

(ภาษาอังกฤษ) Antibody Response in People living with HIV: Comparing CD4 T-cell Levels >200 vs. ≤200 cells/mm^3^ After mRNA COVID-19 Vaccine Booster

1. **ชื่อคณะผู้วิจัย (Investigators)**

**ผู้วิจัยหลัก** นายแพทย์ นภนต์ หิรัญบูรณะ

นิสิตหลักสตูรวิทยาศาสตรมหาบัณฑิต สาขาวิชาอายุรศาสตร์ (โรคติดเชื้อ) คณะแพทยศาสตร์ จุฬาลงกรณ์มหาวิทยาลัย

**ผู้วิจัยร่วม** ผู้ช่วยศาสตราจารย์นายแพทย์ โอภาส พุทธเจริญ

สาขาวิชาอายุรศาสตร์ (โรคติดเชื้อ) คณะแพทยศาสตร์ จุฬาลงกรณ์มหาวิทยาลัย

1. **ความสำคัญและที่มาของปัญหาการวิจัย (Background and Rationale)**

เนื่องจากการติดเชื้อติดเชื้อเอชไอวีเป็นปัจจัยเสี่ยง(independent risk factor) ในการเพิ่มโอกาสการเป็นโรคโควิด-19 แบบรุนแรง และเพิ่มอัตราการเสียชีวิตของผู้ป่วย^1,2,6^ การป้องกันโรคระดับปฐมภูมิหรือการฉีดวัคซีนป้องกันโควิด-19 นั้นมีหลักฐานในการช่วยลดระดับความรุนแรงของโรคโควิด-19ได้เมื่อมีการติดเชื้อ ในทุกสายพันธุ์ อย่างไรก็ดี โอกาสการติดเชื้อนั้นยังมีปัจจัยอื่นๆนอกเหนือจากวัคซีนอีกมาก เช่นปริมาณเชื้อที่ได้รับหรือสัมผัส, ตัวเชื้อโควิดมีการกลายพันธุ์ที่โปรตีนหนามที่ผิวไวรัส (Spike protein) ทำให้หลีกหนีภูมิคุ้มกันทางน้ำเหลือง (humoral immunity) และ ปัจจัยการมีภูมิป้องกันอยู่เดิมของผู้ที่สัมผัสเชื้อเองทั้งภูมิคุ้มกันทางน้ำเหลืองในการจับไวรัสแบบจำเพาะ (neutralized antibody) ยังมีบทบาทของภูมิคุ้มกันอื่นๆในการป้องกันการติดเชื้อ เช่น ระดับ IgA , และ ภูมิคุ้มกันโดยกลไกลทางเซลล์โดยเฉพาะการตอบสนองของเม็ดเลือดขาวชนิด T cell

การวัดประสิทธิภาพของวัคซีนโควิด-19 แบบ primary series ผ่านการศึกษาการตอบสนองทางภูมิคุ้มกัน (Anti RBD IgG) ในกลุ่มผู้ป่วยเอชไอวีหลังฉีดวัคซีนโควิด-19 พบว่ามีการตอบสนองที่น้อยกว่าคนทั่วไป และเทียบเคียงกับกลุ่มผู้ป่วยที่มีภูมิคุ้มกันต่ำอื่นๆ เช่น กลุ่มผู้ป่วยโรค autoimmune, โรคมะเร็งที่ได้รับยาเคมีบำบัด แต่ก็ตอบสนองดีกว่ากลุ่มผู้ป่วยปลูกถ่ายอวัยวะและ ปลูกถ่ายไขกระดูก และการที่ระดับ Anti RBD IgG หลังฉีดวัคซีนขึ้นในระดับปานกลางนั้น ในรายละเอียดแล้วมีความสามารถของแอนติบอดีในการจับแบบจำเพาะ (neutralization level) ที่affinity น้อยกว่า กลุ่มที่มีค่า Anti RBD IgG ที่ขึ้นสูง^3^ ซึ่งกลุ่มผู้ป่วยเอชไอวีในการศึกษาส่วนใหญ่มีการตอบสนองของแอนติบอดีระดับปานกลาง

เมื่อดูในกลุ่มผู้ติดเชื้อเอชไอวีเองแล้ว พบว่าข้อมูลการตอบสนองของภูมิคุ้มกันหลังฉีดวัคซีนโควิด-19 ชนิด mRNA แบบ primary series มีความสัมพันธ์กับ จำนวนเม็ดเลือดขาวชนิดCD4 ซึ่งอาจเป็นการบอกสถานะควบคุมไวรัสด้วยยาต้าน และภูมิคุ้มกันของผู้ป่วย โดยกลุ่มที่มี CD4 สูงกว่ามีระดับรวมของ Anti RBD IgG สูงกว่ารวมถึงมีความสามารถในการ neutralization ที่ดีกว่า^4^

จึงเป็นที่มาของการศึกษานี้ในการหาข้อมูลการตอบสนองทางภูมิคุ้มกันภายหลังการฉีดวัคซีนmRNA เข็มกระตุ้น ในผู้ติดเชื้อเอชไอวีไทยที่มีระดับเม็ดเลือดขาวชนิดCD4 น้อยกว่า 200 เซลล์/มม.^3^ เทียบกับกลุ่มที่มี CD4 มากกว่า 200 เซลล์/มม.^3^  โดยมีการวัดการตอบสนองทั้งในแง่ระดับรวมของภูมิอิมมูโนโกลบูลิน(immunoglobulin levels), การตรวจภูมิคุ้มกันแบบจับเฉพาะ(neutralizing antibodies (nAb)) ในสายพันธุ์ใหม่ของโควิด-19 ที่กำลังมีการระบาด เช่น สายพันธุ์ XBB, BA.2.75 ความสัมพันธ์ของระดับรวมของภูมิอิมมูโนโกลบูลินและการจับเฉพาะ รวมถึง การมองหาปัจจัยอื่นๆของตัวผู้ติดเชื้อเอชไอวี ที่อาจเกี่ยวข้องกับการตอบสนองของวัคซีน

1. **ทบทวนวรรณกรรมที่เกี่ยวข้อง (Review of the Related Literatures)**

ข้อมูลปัจจุบันในกลุ่มผู้ติดเชื้อเอชไอวี พบว่าการที่มีระดับเม็ดเลือดขาวชนิดCD4 ต่ำ, อัตราส่วน CD4/CD8 ที่ต่ำหรือมีระดับไวรัสเอชไอวีในเลือดก่อนฉีดวัคซีนที่สูง ส่งผลต่อการตอบสนองทางภูมิคุ้มกันที่น้อยลง ทั้งหลังการติดเชื้อโควิด19 และ หลังการฉีดวัคซีน ในแง่อัตราการตรวจพบภูมิอิมมูโนโกลบูลินที่จำเพาะต่อเชื้อหลังฉีดวัคซีน(seroconversion rate) และระดับรวมของภูมิอิมมูโนโกลบูลิน (Total Anti RBD Ig levels) ก็ลดลงด้วยอย่างมีนัยยะสำคัญ เช่นกัน^3-5^

ทางทฤษฎีคาดว่าเกิดจากการที่ระดับ CD4 ต่ำ นอกจากในแง่จำนวนแล้ว, CD4ที่มีประสิทธิภาพไม่ดีจากการติดเชื้อเอชไอวี ทำให้การนำเสนอ Antigen ของ Antigen presenting cell ทำได้ไม่มีประสิทธิภาพ มีความล้าของ adaptive immune system และกระตุ้นmemory B/ T cell ได้ลดลง ส่งผลต่อการตอบสนองทางภูมิที่แย่ลง^17^

Haidar G และคณะ^3^ ศึกษา prospective study ของ humoral response หลังได้วัคซีนโควิด-19 (adenovirus vaccine, mRNA vaccine ที่อย่างน้อย 14 วันก่อนเข้าร่วมการศึกษา) ในกลุ่มผู้ป่วยที่มีภูมิคุ้มกันบกพร่องจากสาเหตุต่างๆทั้งสิ้น 1,099 คน เทียบกับ healthcare workers 172 คน โดย primary outcome เป็นการดู seropositivity ของ Anti-RBD IgG และ secondary outcome ดูการเปรียบเทียบของระดับ antibody กับ pseudo virus neutralization levels โดยมีกลุ่มเปรียบเทียบคือ healthcare workers ที่มีอัตรา seropositiveที่ 92.4%(95% CI, 87.4–95.9) กลุ่มผู้ติดเชื้อเอชไอวี 94 คน มีอัตรา seropositive 79.8% (95% CI, 70.2–87.4, P < .01)
และทั้งหมด 52.7%ของ กลุ่มที่ seropositive มีการตอบสนองของ antibody ในระดับที่ต่ำ ซึ่งประชากรผู้ป่วยเอชไอวีในการศึกษานี้ 97% มีการควบคุมไวรัสเอชไอวีได้ (viral suppressed) และ87% มี CD4 มากกว่า 200 เซลล์/มม.^3^ การศึกษานี้ทำให้เห็นว่ากลุ่มผู้ป่วยเอชไอวี มี humoral response หลังฉีดวัคซีนโควิดน้อยกว่าคนทั่วไปอย่างมีนัยยะสำคัญ แม้การศึกษาจะมีข้อจำกัดที่เวลาการเจาะเลือดตรวจภูมิ หลังฉีดวัคซีนไม่เท่ากันในแต่ละบุคคล

Antinori A และคณะ^4^ ศึกษา prospective study ของการตอบสนองทางภูมิคุ้นกันของผู้ติดเชื้อเอชไอวีหลังฉีดวัคซีนโควิด-19 ชนิด mRNA แบบ primary series 2 เข็ม โดยแบ่งเป็น 3 กลุ่มตามระดับ CD4 คือ (poor CD4 recovery, PCDR: <200 เซลล์/มม.^3^; intermediate CD4 recovery, ICDR: 200–500 เซลล์/มม.^3^; high CD4 recovery, HCDR: >500 เซลล์/มม.^3^) เทียบกับ healthcare workers (HCWs) ทีเป็น control และวัดระดับ anti RBD IgG, neutralization antibody, IFN gamma release ที่ช่วงเวลาก่อนฉีดวัคซีน, หลังฉีดเข็มแรก และหลังฉีดเข็ม 2 เป็นเวลา 1 เดือน แม้แต่ละกลุ่มที่แบ่งผู้ป่วยตามระดับ CD4 จะมีจำนวนไม่เท่ากัน และ ลักษณะของแต่ละกลุ่มต่างกันมาก เช่นในกลุ่มที่มี CD4 ต่ำกว่า 200 เซลล์/มม.^3^ ส่วนใหญ่ควบคุมระดับไวรัสเอชไอวีไม่ได้ และไม่ได้รับยาต้านไวรัสเอชไอวี มีระยะเวลาการเป็น HIV มานาน แต่การศึกษานี้ก็ทำให้เห็นภาพรวมของการตอบสนองทางภูมิคุ้มกัน เมื่อใช้ Wilcoxon sign-rank test เปรียบเทียบความแตกต่างของผลลัพธ์ระหว่างแต่ละช่วงเวลาการตรวจภูมิทั้งสามครั้ง และใช้ Kruskal- Wallis test เพื่อดูความแตกต่างของทั้งสามกลุ่ม CD4 พบว่า ในผู้ติดเชื้อเอชไอวีทั้งสิ้น 166คน หลังฉีดวัคซีนเข็มที่สอง1 เดือน ตรวจพบ Anti-RBD IgG 86.7% ในกลุ่ม PCDR, 100% ในกลุ่ม ICDR และ 98.7% ในกลุ่ม HCDR ส่วนมาตรวัดในแง่ neutralization คือ neutralizing titre ≥1:10 นั้นพบ 70.0%, 88.2%, และ93.1% ตามลำดับ เมื่อเปรียบเทียบโดยใช้กลุ่ม HCDR เป็นมาตรฐาน ในกลุ่ม PCDR มีทุกมาตรวัดของการตอบสนองทางภูมิคุ้มกัน(anti-RBD, neutralization antibody และ IFN gamma response) ที่แย่กว่า และเมื่อเปรียบเทียบโดยใช้กลุ่ม HCWs เป็นมาตราฐานแล้ว ในกลุ่ม ICDR (CD4 200–500 เซลล์/มม.^3^) มีเพียงการตอบสนองของ Anti RBD IgG ที่ต่ำกว่าเพียงมาตรวัดเดียวเท่านั้น ในขณะทีกลุ่ม HCDR เทียบเคียงกับ HCWs ในทุกมาตรวัด

ผลไปในทางเดียวกันกับการศึกษาของ Hassold N และคณะ^5^ ที่เป็น retrospective study เก็บข้อมูลจากผู้ติดเชื้อเอชไอวี 105 คน ระยะเวลาตั้งแต่ 8 ถึง 150 วัน (มัธยฐานที่ 73 วัน) หลังจากได้วัคซีน primary series เข็มที่สอง โดยแบ่งแป็นกลุ่มผู้ป่วยตามระดับ CD4 เหมือนกันกับ การศึกษาของ Antinori A และคณะ^4^ พบว่า กลุ่มที่มี CD4 น้อยกว่า 500 เซลล์/มม.^3^ โดยเฉพาะน้อยกว่า 200 เซลล์/มม.^3^ มี seroconversion rates ที่ต่ำกว่า และ ระดับ Antibodyต่อ spike protein ที่ต่ำกว่า เมื่อเทียบกับกลุ่มที่มี CD4 มากกว่า 500 เซลล์/มม.^3^

เมื่อดูจากทั้งสามการศึกษาแล้ว^3-5^ พบว่าหลังจากฉีดวัคซีนทั้งชนิด mRNA, วัคซีนที่ใช้ไวรัสเป็นพาหะ(Viral vector) และวัคซีนเชื้อตาย(Inactivated virus) ในกลุ่มผู้ป่วยเอชไอวีหลังแบ่งกลุ่มตามระดับเม็ดเลือดขาวชนิดCD4^3-5^ พบว่ามีอัตราการตรวจพบภูมิอิมมูโนโกลบูลินต่อเชื้ออย่างมีนัยยะสำคัญหลังฉีดวัคซีน(seroconversion rate)ที่ลดลงในกลุ่มที่มี CD4 ต่ำ และเมื่อได้มีการทำ systematic review โดย Helen M Chun และคณะ^14^ จาก 28 การศึกษาช่วง 1 มกราคม พ.ศ.2564 ถึง 31 มีนาคม พ.ศ.2565 จากแหล่งข้อมูล MEDLINE, Embase, Global Health (OVID), Cochrane Library, CINAHL, Scopus, WHO Global COVID Literature ที่ดูการตอบสนองทางภูมิคุ้มกันหลังฉีดวัคซีนโควิด-19 ในแง่ seroconversion ผู้ติดเชื้อเอชไอวี พบว่า ผู้ติดเชื้อที่มีระดับ CD4 และ สัดส่วน CD4/CD8^7^ ที่สูง และควบคุมระดับไวรัสได้ (suppressed HIV VL) มีการตอบสนองทางภูมิคุ้มกันที่ดีกว่า
แต่ว่าในแต่ละการศึกษาเองมีจุดบกพร่องที่สำคัญบางประการแตกต่างกันไป เช่นการมีกลุ่มผู้ป่วยที่มีระดับเม็ดเลือดขาวชนิด CD4 <200 เซลล์/มม.^3^ จำนวนน้อย^3^, ช่วงเวลาที่ทำการวัดระดับอิมมูโนโกลบูลินในเลือดหลังฉีดวัคซีนต่างกัน, วัคซีนที่ใช้มีทั้งเข็มกระตุ้นชนิด mRNA (mRNA-booster) และวัคซีนแรกเริ่ม(primary series), การไม่ได้มีการวัดภูมิคุ้มกันจำเพาะ (neutralizing antibodies (nAb)) ต่อสายพันธุ์ย่อยของไวรัสโควิด-19 โดยใช้วิธีการจำลองการจับของแอนติบอดีกับเชื้อไวรัสแบบตกตะกอน (neutralized) ในหลอดทดลองด้วยวิธี ELISA (Standardized pseudovirus neutralization assays หรือเรียกว่า surrogate virus neutralisation test (sVNT))^9^ หรือ อีกวิธีหนึ่งคือการจำลองการตกตะกอน(neutralized) ของไวรัสเทียมที่มีโปรตีนหนามของโควิด-19 (เรียกว่า SARS- CoV-2 Spike (S)-Pseudo typed Virus Neutralization Assay (PsVNA))^10,11^ และ การไม่ได้วัดการตอบสนองทางเม็ดเลือดขาวชนิดT cell (T cell response) และอิมมูโนโกลบูลินเอ (IgA level) ซึ่งอาจเป็นปัจจัยป้องกันการติดเชื้อหลังฉีดวัคซีน

ในภาพรวมทำให้เห็นข้อมูลว่าในกลุ่มผู้ติดเชื้อเอชไอวีที่มี ระดับเม็ดเลือดขาวชนิดCD4 น้อยกว่าหรือเท่ากับ 200 เซลล์/มม.^3^ มีการตอบสนองของภูมิคุ้มกันของวัคซีนที่น้อยกว่ากลุ่มที่มี CD4 มากกว่า 200 เซลล์/มม.^3^

สถานการณ์ในปัจจุบันที่โควิดเป็นเชื้อประจำถิ่น ผู้คนส่วนใหญ่ได้ผ่านการติดเชื้อและได้รับวัคซีนมาแล้วและมีอาการแสดงที่หลากหลายตั้งแต่ไม่มีอาการไปจนถึงอาการรุนแรง จึงมีภูมิคุ้มกันแบบผสมและทำให้เกิดการจดจำและมีการปรับตัวเติบโตของบีเซลล์ (memory B-cell, B-cell maturation) ทำให้เกิดการสร้างแอนติบอดี ที่มีความจำเพาะต่อสายพันธุ์ใหม่ตามช่วงเวลานั้นๆได้เพิ่มมากขึ้นหลังการฉีดวัคซีนกระตุ้น

ปัจจุบันได้มีการพัฒนาวัคซีนแบบ 2 สายพันธุ์ (bivalent vaccine) ประกอบด้วย mRNA ของสายพันธุ์ดั้งเดิม และสายพันธุ์โอมิครอนอย่างละครึ่งที่ผ่านการศึกษาเฟส2แล้ว^12^ เช่น วัคซีนแบบ 2 สายพันธุ์ของ Moderna สำหรับผู้ใหญ่ขนาด 50 ไมโครกรัม แบ่งเป็นสายพันธุ์ดั้งเดิม 25 ไมโครกรัม และสายพันธุ์โอมิครอน( สายพันธุ์ย่อย BA.1 หรือ BA.4/BA.5) 25 ไมโครกรัม แนวคิดคือมีการกระตุ้น memory B cell ที่มีอยู่ด้วยสายพันธุ์ดั้งเดิม และ กระตุ้นให้สร้าง B-cell ที่มีภูมิต่อสายพันธุ์ใหม่ไปพร้อมๆกัน จึงน่าจะเกิดปริมาณภูมิคุ้มกันจำเพาะ(neutralizing antibodies (nAb)) จากการเป็นวัคซีนเข็มกระตุ้นที่เพิ่มมากขึ้น และมีประโยชน์ในการเพิ่มความสามารถของantibodyในการจับกับสายพันธุ์อื่นๆผ่านกลไกล cross-reactivity ด้วย ข้อมูลจากการศึกษาในเฟส 2^12^  พบว่าเมื่อดูการตอบสนองทางภูมิคุ้มกันทางน้ำเหลือง (humoral immunity) เปรียบเทียบระดับภูมิคุ้มกันหลังฉีดวัคซีนเข็มกระตุ้นที่ 2 (เข็มที่ 4) ระหว่างวัคซีนแบบไบวาเลนต์ (bivalent vaccine) สายพันธุ์ดั้งเดิม และ BA.1 เทียบกันกับ วัคซีน m-RNA รุ่นเก่า (สายพันธุ์เดียว) พบว่าในวัคซีนแบบไบวาเลนต์ กระตุ้นภูมิคุ้มกันได้สูงกว่า กล่าวคือ ระดับรวมของภูมิอิมมูโนโกลบูลิน (immunoglobulin levels) ต่อ BA.1 (*เทียบเป็น geometric mean level) ในวัคซีนแบบไบวาเลนต์ 2,372.4 หน่วย วัคซีน m-RNA รุ่นเก่า 1,473.5 หน่วย ภูมิคุ้มกันต่อ BA.4/BA.5 ซึ่งเป็นสายพันธุ์หลักที่มีการระบาดในช่วงปลายปี พ.ศ.2565 : วัคซีนแบบไบวาเลนต์ 727.4 หน่วย ส่วนวัคซีน m-RNA รุ่นเก่า 492.1 หน่วย สังเกตว่าระดับการกระตุ้นภูมิต่อสายพันธุ์ BA.4/BA.5 ของวัคซีนทั้งคู่น้อยกว่าสายพันธุ์ BA.1 ซึ่งเป็นสายพันธุ์ที่อยู่ในวัคซีนแบบไบวาเลนต์ นอกจากนี้ ภูมิคุ้มกันต่อสายพันธุ์อัลฟา, เบตา, แกมมา และเดลตาของวัคซีน ยังสูงกว่าอีกด้วย แต่ในข้อมูลล่าสุดจาก Qian Wang และคณะ^15^ ศึกษาการตอบสนองหลังฉีด bivalent covid vaccineในแง่ความสามารถในการสร้างแอนติบอดีที่สามารถจับอย่างจำเพาะ(neutralizing antibodies (nAb)) กับสายพันธุ์ต่างๆ รวมถึงสายพันธุ์ BA.4–BA.5, BA.4.6, BA.2.75, และ BA.2.75.2 ที่ที่กำลังระบาดพบว่าไม่มีความแตกต่างอย่างมีนัยยะสำคัญ ระหว่างกลุ่มที่ฉีด mRNA vaccine (monovalent) 4 เข็มและ กลุ่มที่ได้ bivalent-booster เป็นเข็มที่ 4 อย่างไรก็ตามกลุ่มประชากรที่ศึกษายังมีกลุ่มละ 20 คนเท่านั้น และไม่ได้รวมกลุ่มที่มีภูมิคุ้มกันบกพร่องเข้ามาร่วมศึกษา

ในช่วงเวลาการศึกษาที่ประชากรมีภูมิคุ้มกันต่อโควิดทั้งจากการฉีดวัคซีน และภายหลังการติดเชื้อ จึงเป็นที่น่าสนใจว่าการตอบสนองทางภูมิคุ้มกันของผู้ติดเชื้อเอชไอวีหลังฉีดวัคซีน mRNA เข็มกระตุ้น ในกลุ่มที่มีระดับเม็ดเลือดขาวชนิดCD4 น้อยกว่า 200 เซลล์/มม^3^ เมื่อเทียบกับกลุ่มที่มี CD4 มากกว่า 200 เซลล์/มม^3^ มีความสามารถในการสร้างแอนติบอดีที่สามารถจับอย่างจำเพาะ(neutralizing antibodies (nAb)) กับสายพันธุ์ที่กำลังระบาดขณะมีการให้ฉีดวัคซีนดังกล่าวมากน้อยเพียงใด โดยใช้วิธีการจำลองการจับของแอนติบอดีกับเชื้อไวรัสแบบตกตะกอน (neutralized) ในหลอดทดลองด้วยวิธี ELISA (surrogate virus neutralisation test (sVNT)) และการจับแบบจำเพาะนั้นมีความเกี่ยวข้องกับระดับรวมของภูมิอิมมูโนโกลบูลิน (Anti-RBD total Ig levels) หรือไม่

1. **คำถามของการวิจัย (Research questions)**

**คำถามหลัก (Primary research question)** กลุ่มผู้ป่วยที่มีระดับเม็ดเลือดขาวชนิดCD4 น้อยกว่าหรือเท่ากับ 200 เซลล์/มม^3^ จะมีการตอบสนอง ทางแอนติบอดี ในแง่ระดับรวมของภูมิอิมมูโนโกลบูลิน (Anti-RBD total Ig levels) ที่ 4 สัปดาห์หลังฉีด mRNA วัคซีนเข็มกระตุ้น น้อยกว่า เมื่อเทียบกับ กลุ่มที่มี CD4 มากกว่า 200 เซลล์/มม^3^ หรือไม่

**คำถามรอง (Secondary research question)**

- กลุ่มผู้ป่วยที่มีระดับเม็ดเลือดขาวชนิดCD4 น้อยกว่าหรือเท่ากับ 200 เซลล์/มม.^3^ จะมีการตอบสนองทางภูมิคุ้มกันแบบจับจำเพาะ(neutralizing antibodies (nAb)) ที่ดูจากระดับเปอร์เซ็นต์การจับเพื่อตกตะกอนของ sVNT (inhibition level) ต่อสายพันธุ์ใหม่ของไวรัสโควิด-19 เช่น XBB, BA.2.75 และ สายพันธุ์ดั้งเดิม หลังได้รับ mRNA วัคซีนเข็มกระตุ้น~~ที่สอง~~ที่ 4 สัปดาห์ น้อยกว่า กลุ่มที่มี CD4 มากกว่า 200 เซลล์/มม^3^ หรือไม่
- ความเกี่ยวข้องระหว่างระดับ รวมของภูมิอิมมูโนโกลบูลิน (Anti-RBD total Ig levels) ที่ 4 สัปดาห์หลังฉีด mRNA วัคซีนเข็มกระตุ้น และ ระดับเปอร์เซ็นต์การจับเพื่อตกตะกอนของ sVNT (inhibition level) ในกลุ่มผู้ป่วยที่มีระดับเม็ดเลือดขาวชนิดCD4 น้อยกว่าหรือเท่ากับ 200 เซลล์/มม.^3^ จะมีความเกี่ยวข้องน้อยกว่า กลุ่มที่มี CD4 มากกว่า 200 เซลล์/มม.^3^ หรือไม่
- ค่าการเพิ่มขึ้นของระดับรวมของภูมิอิมมูโนโกลบูลิน (Anti-RBD total Ig levels) ที่ 4 สัปดาห์เทียบกับก่อนฉีดวัคซีน mRNA เข็มกระตุ้น ในกลุ่มผู้ป่วยที่มีระดับเม็ดเลือดขาวชนิดCD4 น้อยกว่าหรือเท่ากับ 200 เซลล์/มม.^3^ จะมีค่าน้อยกว่า กลุ่มที่มี CD4 มากกว่า 200 เซลล์/มม.^3^ หรือไม่

1. **วัตถุประสงค์ของการวิจัย (Objectives)**

**วัตถุประสงค์หลัก** (Primary objective) : ประเมินการตอบสนองทางภูมิคุ้มกันในแง่ระดับรวมของภูมิอิมมูโนโกลบูลิน (Anti-RBD total Ig levels) ที่ 4 สัปดาห์หลังได้รับ mRNA วัคซีนเข็มกระตุ้น ในกลุ่มผู้ป่วยที่มีระดับเม็ดเลือดขาวชนิดCD4 น้อยกว่าหรือเท่ากับ 200 เซลล์/มม.^3^ เทียบกับ กลุ่มที่มี CD4 มากกว่า 200 เซลล์/มม.^3^

**วัตถุประสงค์รอง** (secondary objective) : เพื่อศึกษา การตอบสนองทางภูมิคุ้มกันโดยวัดภูมิคุ้มกันแบบจำเพาะ(neutralizing antibodies (nAb)) ต่อสายพันธุ์ใหม่ของไวรัสโควิด-19 เช่น XBB, BA.2.75 ที่ 4 สัปดาห์หลังได้รับ mRNA วัคซีนเข็มกระตุ้น
เปรียบเทียบระหว่างกลุ่มผู้ป่วยที่มีระดับเม็ดเลือดขาวชนิดCD4 น้อยกว่าหรือเท่ากับ 200 เซลล์/มม.^3^ เทียบกับ กลุ่มที่มี CD4 มากกว่า 200 เซลล์/มม.^3^ โดยใช้วิธีการจำลองการจับของแอนติบอดีกับเชื้อไวรัสแบบตกตะกอน (neutralized) ในหลอดทดลองด้วยวิธี ELISA (Standardized pseudovirus neutralization assays หรือเรียกว่า surrogate virus neutralization test (sVNT)) และดูความสัมพันธ์ของ ระดับเปอร์เซ็นต์การจับเพื่อตกตะกอนของ sVNT (inhibition level) กับระดับรวมของภูมิอิมมูโนโกลบูลิน (Anti-RBD total Ig levels) รวมถึง ค่าการเพิ่มขึ้นของระดับรวมของภูมิอิมมูโนโกลบูลิน (Anti-RBD total Ig levels) ที่ 4 สัปดาห์เทียบกับก่อนฉีดวัคซีน mRNA เข็มกระตุ้น

1. **สมมติฐาน (Hypothesis)**

กลุ่มผู้ป่วยที่มีระดับเม็ดเลือดขาวชนิดCD4 น้อยกว่าหรือเท่ากับ 200 เซลล์/มม.^3^ จะมีการตอบสนอง ทางภูมิคุ้มกันในแง่ระดับรวมของภูมิอิมมูโนโกลบูลิน (Anti-RBD total Ig levels) หลังได้รับ mRNA วัคซีนเข็มกระตุ้น น้อยกว่า เมื่อเทียบกับกลุ่มที่มี CD4 มากกว่า 200 เซลล์/มม.^3^  เทียบกันเป็น geometric mean ratio (GMR) น้อยกว่า 0.4 ขึ้นไป

1. **กรอบแนวความคิดในการวิจัย (Conceptual Framework)**

**The second booster dose of COVID-19 mRNA vaccine**

- **Type: Bivalent/ Monovalent**
- **Different brand (amount of mRNA): Pfizer–BioNTech ((30 μg), Moderna(50 μg)**
- **administration ID or IM**

**Anti RBD immunoglobulin G levels at 4 weeks**

**After a booster dose of mRNA COVID-19 vaccine**

**Two groups of PLWH according to CD4 level**

- CD4 > 200 cells/mm^3^
- CD4 ≤ 200 cells/mm^3^

**Confounders**

**Other patient factors**

**PLWH(People living with HIV);
Comorbidities, time since diagnosed HIV, viral suppressed duration, ARV regimen, History of Opportunistic infection, History of virologic failure, Immunosuppressive drugs use**

**New COVID-19 infection in 4 weeks**

**Any vaccination 2 weeks before COVID-19 vaccine second booster dose**

**Anti RBD IgG test validity, consistency**

**Previous COVID-19 vaccination factors**

- **Previous primary series vaccine**
  (type of vaccine, timing, administration ID or IM)
- **Previous first mRNA booster vaccine** **(≥3 months earlier).**

(type of vaccine, timing, administration ID or IM)

- **Duration from last COVID-19 vaccination**

**Previous COVID-19 infection factor (in previous 3 months)**

1. **คำสำคัญ (Key words)**immunogenicity; Antibody response; SARS-CoV-2 ; Anti–SARS-CoV-2 vaccine; HIV/AIDs; CD4 level; Surrogate virus neutralization test
2. **การให้คำนิยามเชิงปฏิบัติที่จะใช้ในงานวิจัย**

- ผู้เข้าร่วมวิจัยที่สงสัยว่าเคยติดเชื้อโควิด-19 ในช่วง 3 เดือน นิยามคือ ผู้เข้าร่วมวิจัยที่เคยมีอาการทางระบบทางเดินหายใจที่เข้าได้กับการติดเชื้อไวรัส และตรวจ ATK (ตรวจที่สถานพยาบาลหรือไม่ก็ได้) หรือ nasal/ nasopharyngeal swab PCR for SARS-CoV2 ยืนยันแล้วให้ผล positive ในช่วงเวลาภายใน 3 เดือนก่อนหน้าวันเข้าร่วมการวิจัย
- ผู้ติดเชื้อเอชไอวีควบคุมระดับไวรัสในเลือดได้ (suppressed viral load) นิยามคือ ผู้ติดเชื้อเอชไอวีที่มีผลHIV viral load ภายใน 1 ปี เป็น undetectable (<20 copies/ml)
- ผู้ติดเชื้อเอชไอวี ที่ควบคุมระดับไวรัสในเลือดไม่ได้ (virologic failure) (> 200 copies/ml)
- ผู้ติดเชื้อเอชไอวีที่ได้รับยาต้านไวรัสอย่างต่อเนื่อง นิยามคือ มีการตรวจติดตามและรับยาต้านไวรัสเป็นเวลามากกว่า 6 เดือน

1. **รูปแบบการวิจัย Research Design​:** Prospective observational cohort study
2. **ระเบียบวิธีการวิจัย (Research Methodology)**

**ประชากร (Population):** ผู้ติดเชื้อเอชไอวีในคลินิกผู้ป่วยนอกอายุมากกว่า 18 ปี ที่มีสถานะเหมาะสมกับการฉีดวัคซีนโควิด-19 ชนิด mRNA เข็มกระตุ้น

**เป้าหมาย (Target Population):** ผู้ติดเชื้อเอชไอวีในคลินิกผู้ป่วยนอกอายุมากกว่า 18 ปี ที่ได้รับยาต้านไวรัสอย่างต่อเนื่อง, ไม่มี virologic failure และมีระดับเม็ดเลือดขาวชนิดCD4 น้อยกว่าหรือเท่ากับ 200 เซลล์/มม^3^

**ประชากรกล่มควบคุม (Control Population):** ผู้ติดเชื้อเอชไอวีในคลินิกผู้ป่วยนอก ได้รับยาต้านไวรัสอย่างต่อเนื่อง, ไม่มี virologic failure และมีระดับเม็ดเลือดขาวชนิดCD4 มากกว่า 200 เซลล์/มม^3^

**วิธีการเข้าถึงอาสาสมัคร (Approach to participant**): เข้าถึงผู้ติดเชื้อเอชไอวีที่เข้ามารับการตรวจที่คลินิกโรคติดเชื้อ ประญัติ ลักษณะพุกก์ อาคาร ภปร ชั้น 14 โรงพยาบาลจุฬาลงกรณ์ และ HIV-NAT หรือ ศูนย์ประสานความร่วมมือระหว่างไทย-ออสเตรเลีย-เนเธอร์แลนด์ เพื่อการศึกษาวิจัยด้านโรคเอดส์ ภายใต้ศูนย์วิจัยโรคเอดส์ สภากาชาดไทยผ่านทางการติดประกาศรับสมัคร และโทรศัพท์สอบถามความสนใจจากผู้ป่วย

**เกณฑ์การคัดเลือกอาสาสมัครเข้าร่วมโครงการวิจัย (Inclusion criteria)**

1. ผู้ติดเชื้อเอชไอวีในคลินิกผู้ป่วยนอกอายุมากกว่า 18 ปี ที่ควบคุมระดับไวรัสในเลือดได้ (suppressed viral load)
2. ได้รับยาต้านไวรัสอย่างต่อเนื่องและเป็นสูตรเดิมอย่างน้อย 3 เดือน
3. เคยได้รับวัคซีนโควิด-19 มาก่อนหน้า ขั้นต่ำเป็น primary series ชนิดใดก็ได้ 2 เข็ม โดยระยะเวลาที่ฉีดเข็มล่าสุด เป็นเวลามากกว่าหรือเท่ากับ 3 เดือน นับจากเข้าร่วมการศึกษา

**เกณฑ์การคัดเลือกอาสาสมัครออกจากโครงการวิจัย (Exclusion criteria)**

1. เคยติดเชื้อโควิด-19 ก่อนหน้าในระยะเวลา 3 เดือนก่อนเข้าร่วมการศึกษา (คัดกรองโดยใช้ประวัติและผล ATK)
2. ฉีดวัคซีนชนิดอื่นก่อนหน้าในระยะเวลา 15 วัน
3. มีข้อห้ามในการฉีดวัคซีน หรือ มีประวัติการแพ้วัคซีนโควิด-19 ก่อนหน้า
4. อยู่ในภาวะ high-level immunosuppression ได้แก่ ยากดภูมิคุ้มกันขนาดสูง คือ prednisolone > 20 mg/day, rituximab, TNF alpha blocker, MTX > 0.4 mg/kg/week
5. ได้รับ monoclonal antibody, JAK inhibitor และ convalescent plasma for COVID-19
6. มีโรคแพ้ภูมิตัวเองระยะกำเริบ (active autoimmune disease) และ โรคมะเร็งไม่ว่าจะที่กำลังรักษา

**วิธีการคำนวณขนาดตัวอย่าง (sample size calculation)**
 การวิเคราะห์กําลังการทดสอบ (Power calculation) มีหลักการเพื่อประมาณดังต่อไปนี้
เริ่มต้นจาก ทีมผู้ดำเนินการวิจัยคาดว่าค่าภูมิคุ้มกันในแง่ระดับรวมของภูมิอิมมูโนโกลบูลิน(Anti-RBD total Ig levels) หลังจากการฉีดวัคซีนเข็มกระตุ้น จะมีการกระจายของข้อมูลแบบล็อกปกติ (log normal) ที่มี
ค่าสัมประสิทธิ์ของความแปรปรวน (Coefficient of Variation: CV) อยู่ที่ 4.4 หรือค่าเบี่ยงเบนมาตรฐานทางเรขาคณิต (Geometric standard deviation) อยู่ที่ 1.71 โดยค่า CV นี้ได้มาจากข้อมูลการศึกษาของ Antinori และคณะตีพิมพ์ในวารสาร Clinical Infectious Diseases^4^  ที่เปรียบเทียบการตอบสนองของ Anti-RBD Ig G levels หลังฉีดวัคซีน mRNA แบบ primary vaccination จำนวน 2 โดส ในผู้ติดเชื้อเอชไอวีที่มีระดับเม็ดเลือดขาวชนิดCD4 น้อยกว่า 200 เซลล์/มม^3^ เทียบกับกลุ่มที่มีเม็ดเลือดขาวชนิดCD4 ที่มากกว่า ซึ่งค่า CV นี้ยังใกล้เคียงกับกับ CV ของการเพิ่มขึ้นของแอนติบอดีหลังวัคซีนเข็มกระตุ้นในการศึกษาแบบ cohort ของผู้ป่วยในคลินิกโรคมะเร็งโรงพยาบาลจุฬา ที่ได้รับการรักษาด้วยยาเคมีบำบัดหลากหลายชนิด (CV = 4.2)^13^

ทางทีมผู้ดำเนินการวิจัยต้องการกำลังการทดสอบ (power) 80% เพื่อดูการลดลงของ geometric mean antibody concentration 60% (หรือ geometric mean ratio = 0.4) ในกลุ่มที่มี CD4 ต่ำ เปรียบเทียบกับกลุ่มที่มีมี CD4 สูงเป็นเป็นมาตราฐาน

ขนาดตัวอย่างสำหรับการเปรียบเทียบค่าเฉลี่ย (mean) ระหว่างสองกลุ่มที่มีการแจกแจงที่เท่ากัน (equal allocation ratio)มาจากสมการด้านล่างนี้

$$\frac{\left( Z_{1-\alpha/2}+Z_{1-\beta} \right)^{2}\left( \sigma^{2} \right)}{\Delta^{2}}$$

เมื่อ $Z_{1-\alpha/2}$ คือ critical value for significance มีค่าเท่ากับ 1.96 ที่ 5% significance level

$Z_{1-\beta}$ คือ critical value for power มีค่าเท่ากับ 0.842 at 80% power

$\sigma$ คือ geometric standard deviation, และ $\Delta$ คือ natural log transformed geometric mean ratio สำหรับ กลุ่ม CD4 ต่ำเทียบกับกลุ่ม CD4 สูงเป็นมาตราฐาน (log(0.4) = 0.91).

หลังจากคำนวณแล้ว ต้องใช้ผู้เข้าร่วมวิจัยทั้งหมด 116 คน (แบ่งเป็นกลุ่มละ 58 คน) จึงจะมีกำลังการทดสอบ (power) 80% เพื่อตรวจสอบการลดลงของ geometric mean antibody ที่มากกว่าหรือเท่ากับ 60% โดยมี 2-sided significance level 5% นอกจากนี้ยังใช้โปรแกรม SAS ซึ่ง US FDA มักใช้ในการเพื่อคำนวณขนาดตัวอย่าง ของการศึกษาประสิทธิภาพวัคซีน (มีการแสดงดังภาพด้านล่าง)


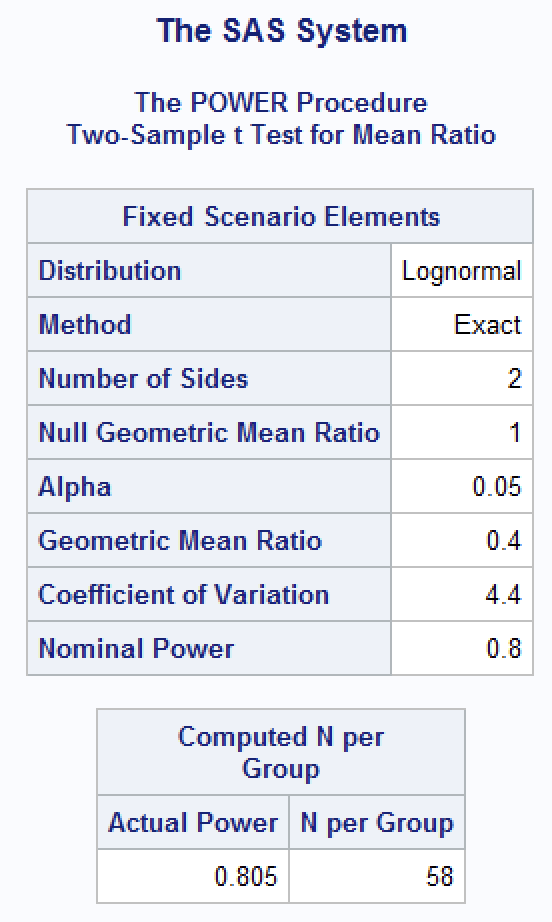
เมื่อกำหนดการประเมิน outcome หลังได้วัคซีนเข็มกระตุ้นเป็นช่วงเวลาห่างกันไม่นาน คิดว่าจำนวนผู้เข้าร่วมการวิจัยที่หายไปจากการทดลองน่าจะมีปริมาณน้อย ดังนั้นปริมาณขนาดตัวอย่างจึงเพิ่มขึ้น 5% เป็นทั้งสิ้น122 คน(กลุ่มละ 61 คน)

รูปที่2 การใช้โปรแกรม Statistical Analysis **System (**SAS)เพื่อคำนวณขนาดตัวอย่าง

**กระบวนการขอความยินยอม (Informed consent process)**

กระบวนการขอความยินยอมจะทำที่โรงพยาบาลจุฬาลงกรณ์และศูนย์วิจัยโรคเอดส์สภากาชาดไทย และ HIV-NAT โดยแพทย์ผู้ทำวิจัยอธิบายขั้นตอนการดำเนินการวิจัย ความเสี่ยงและประโยชน์ ตอบข้อสงสัยจน อาสาสมัครเข้าใจ และให้เวลาตัดสินใจโดยอิสระ ก่อนลงนามให้ความยินยอมเข้าร่วมในการวิจัย

**วิธีการวิจัย/วิธีดำเนินการวิจัย**

1. **การรวบรวมข้อมูล (Data collection)**
2. ทีมผู้ดำเนินการวิจัยเก็บข้อมูลผู้ติดเชื้อเอชไอวีที่มีสถานะเหมาะสมกับการฉีดวัคซีนโควิด-19 ชนิด mRNA เข็มกระตุ้นที่สอง ณ คลินิกผู้ป่วยนอกโรคติดเชื้อ ภปร.ชั้น14 และ ศูนย์วิจัยโรคเอดส์สภากาชาดไทย
3. ประเมินว่าผู้ป่วยเข้าเกณฑ์ inclusion และ exclusion criteria
4. ทีมผู้วิจัยอธิบายให้อาสาสมัครทราบถึงที่มาและขั้นตอนการดำเนินการวิจัยดังนี้
5. ให้อาสาสมัครอ่านใบยินยอมในการเข้าร่วมโครงการวิจัยโดยลงชื่อในใบยินยอมพร้อมลงวันที่ที่ผู้ป่วยลงชื่อ
6. ทีมผู้ดำเนินการวิจัยทำการเก็บข้อมูลดังต่อไปนี้
   - ประวัติข้อมูลส่วนตัว ได้แก่ เพศ อายุ
   - โรคประจำตัวอื่นๆ
   - ระดับเม็ดเลือดขาวชนิดCD4 ล่าสุดภายใน 6 เดือน ในคลินิก และระดับช่วงที่ต่ำที่สุด, อัตราส่วนระหว่างเม็ดเลือดขาวชนิด CD4/CD8 (หากมี)
   - ระยะเวลาตั้งแต่วินิจฉัยการติดเชื้อเอชไอวี
   - สูตรยาต้านไวรัส และระยะเวลาที่ได้ยาต้านไวรัส
   - ระยะเวลาที่ที่ควบคุมระดับไวรัสในเลือดได้ (suppressed viral load)
   - ประวัติการติดเชื้อฉวยโอกาส และการควบคุมไวรัสเอชไอวีในเลือดไม่ได้หลังจากเริ่มรักษา (virologic failure) จากข้อมูลที่มีตั้งแต่เริ่มรับยารักษา
   - ประวัติการฉีดวัคซีนโควิดก่อนหน้า
   - ประวัติการฉีดวัคซีนชนิดอื่นๆ
7. นัดแนะวันเวลาเพื่อเก็บตัวอย่างเลือดชุดแรก(ปริมาตร 15 มิลลิลิตร) เพื่อตรวจ
   - CD4, CBC
   - anti-spike RBD-binding antibody (Anti RBD total Ig)
   - ภูมิคุ้มกันแบบจำเพาะ (neutralizing antibodies) ที่ดูจากระดับเปอร์เซ็นต์การจับเพื่อตกตะกอนของ sVNT (%inhibition level) ต่อสายพันธุ์ใหม่ของไวรัสโควิด-19 เช่น XBB, BA.2.75 และสายพันธุ์ดั้งเดิม
8. ฉีดวัคซีนโควิด-19 ชนิด bivalent mRNA เข็มกระตุ้น ในวันเดียวกันหลังจากเก็บตัวอย่างเลือดชุดแรก และเฝ้าระวังผลข้างเคียงหลังฉีดวัคซีนที่จุดฉีดวัคซีน เป็นระยะเวลา 30 นาที
9. นัดวันเวลาการเก็บตัวอย่างเลือดชุดที่สอง (ปริมาตร 15 มิลลิลิตร) หลังจากฉีดวัคซีนเป็นเวลา 28 วัน (Anti RBD total Ig และ sVNT (%inhibition level))
10. ระหว่างรอตรวจเลือดชุดที่สอง ทีมผู้ดำเนินการวิจัยโทรติดตามผู้ป่วยทุก 1 สัปดาห์เพื่อตรวจสอบอาการที่สงสัยการติดเชื้อโควิด-19 รวมถึงอาการที่สงสัยผลข้างเคียงจากวัคซีน
11. สำหรับผู้ป่วยที่มีอาการสงสัยการติดเชื้อโควิด-19 และผล ATK เป็นบวก หากมีความเสี่ยงต่อการเกิดโรครุนแรง และเข้าเกณฑ์การได้ยาต้านไวรัสโควิด จะติดต่อผู้ป่วยที่มีความเสี่ยงดังกล่าวมารับการตรวจบริการที่โรงพยาบาลจุฬาลงกรณ์
    *ทั้งนี้ผู้ป่วยกลุ่มดังกล่าวจะถูกคัดออกจากการศึกษา
12. ผู้ป่วยที่ไม่มีอาการสงสัยการติดเชื้อโควิด ให้มารับการตรวจเลือดชุดที่สองตามนัด
13. วิเคราะห์ข้อมูลและสรุปรายงานวิจัย

รูปที่1 วิธีดำเนินการวิจัย (Study flow)
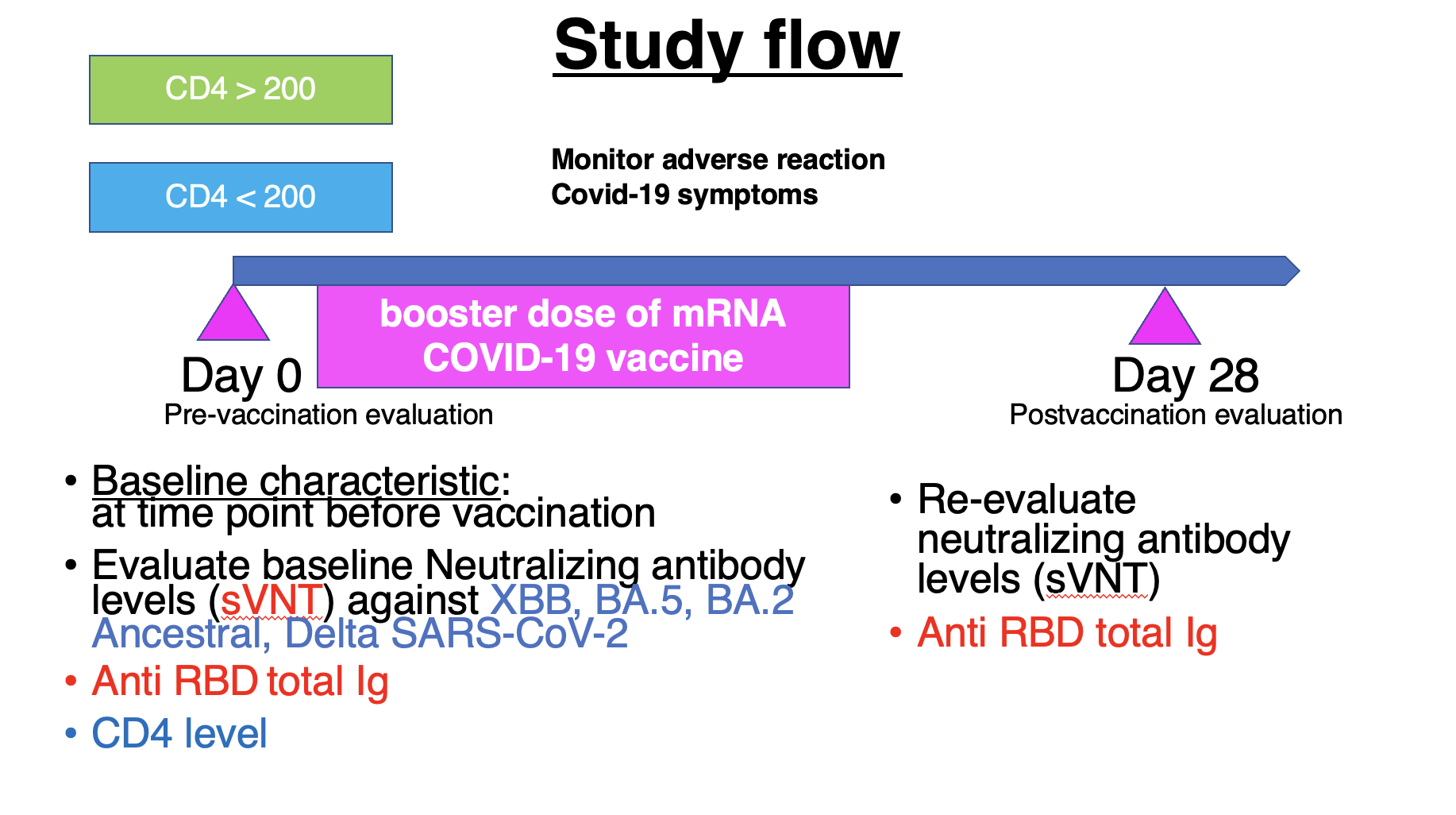


**การตรวจทางห้องปฏิบัติการและการแปลผล**

**การตรวจวัดระดับภูมิคุ้มกันในแง่ระดับรวมของภูมิอิมมูโนโกลบูลิน ต่อโรคติดเชื้อไวรัสโคโรนา 2019
(Anti-Receptor binding domain total immunoglobulin)**
เป็นการหาปริมาณแอนติบอดีจากเซรัมโดยจำเพาะกับ receptor-binding domain (RBD) ที่spike protein ของเชื้อ SARS-CoV-2 (Anti RBD total Ig levels) ด้วยเครื่อง Elecsys Cobas e601 ของบริษัท Roche Diagnostics, Basel, Switzerland ที่ใช้วิธี Electrochemiluminescence mmunoassay (ECLIA) โดยมี limit of detection ของ assayนี้อยู่ที่ 0.4 U/mL

**การแปลผลของ test** : ค่าantibody concentrations ที่มากกว่าหรือเท่ากับ 0.8 U/mL จะแปลผลว่า positive

**การตรวจวัดระดับภูมิคุ้มกันต่อโรคติดเชื้อไวรัสโคโรนา 2019 (SARS-CoV2 neutralizing antibody)**
โดยวิธี Blocking Enzyme-Linked Immunosorbent Assay (ELISA) จากเลือด รายงานผลเป็นแบบ qualitative จาก cPass™ SARS-CoV-2 Neutralization Antibody Detection Kit (GenScript) ซึ่งได้รับการรับรองจาก FDA Emergency Use โดยจะวัดระดับ Antibody ต่อส่วนของ Horseradish peroxidase (HRP) conjugated recombinant SARS-CoV2-RBD fragment (HRP-RBD) และ human ACE2 receptor protein (hACE2) นอกจากนี้ยังส่ง neutralizing antibody ต่อ SARS-CoV2 specific สายพันธุ์ที่ระบาดอยู่ในประเทศไทย หน่วยวัดที่คำนวณได้จะออกมาเป็น Percent signal inhibition โดยจะส่งตรวจที่ห้องปฏิบัติการ คณะแพทยศาสตร์ จุฬาลงกรณ์มหาวิทยาลัย

**การแปลผลของ test** : การไม่มีภูมิคุ้มกัน (Seronegative) ต่อโรคติดเชื้อไวรัสโคโรนา 2019 (SARS-CoV2 Neutralizing Antibody) ตัดที่ผล < 30% Signal Inhibition ^16^

การมีภูมิคุ้มกันถึงระดับที่ป้องกันโรคติดเชื้อไวรัสโคโรนา 2019 ได้ (Sero-protection) หมายถึงการมีภูมิคุ้มกันต่อโรคติดเชื้อไวรัสโคโรนา 2019 (SARS-CoV2 Neutralizing Antibody) ตัดที่ผล ≥ 68% Signal Inhibition ^16^

ส่วนค่า signal inhibition ระหว่าง 30 – 67% ถือว่ามีภูมิคุ้มกันต่อโรคติดเชื้อไวรัสโคโรนา 2019 (SARS-CoV2 Neutralizing Antibody) เช่นกัน แต่ไม่ได้เข้าเกณฑ์การรับรองว่ามี Sero-protection

1. **การวิเคราะห์ข้อมูล (Data analysis)**

สำหรับ primary outcome ซึ่งคือ geometric mean ratio ของ antibody concentrations ทางทีมวิจัยจะใช้ regression model โดยคิดจาก outcome ของ Anti-RBD total Ig levels ที่ 4 สัปดาห์ หลังฉีดวัคซีนเข็มกระตุ้นที่ถูกแปลงเป็น natural log ตามข้อมูลในแต่ละกลุ่ม CD4

ค่าสัมประสิทธิ์(model coefficient) และ95%Confident interval (CI) จะถูกทำการ expotentiated เพื่อให้ได้ค่า geometric mean ratio (GMR) และ 95%CI หากมีการแตกต่างอย่างชัดเจนในข้อมูลพื้นฐาน(group demographic) หรือลักษณะbaseline characteristic ในผู้ป่วยเอชไอวีทั้งสองกลุ่ม ทางทีมวิจัยก็จะจัดทำ adjusted models ต่อไป และการวิเคราะห์ข้อมูลจะ stratified แบ่งผู้ป่วยในแต่ละกลุ่ม CD4 เป็น 1.กลุ่มที่เคยมีการติดเชื้อโควิดมาก่อนระยะเวลา 3 เดือนก่อนฉีดวัคซีน mRNA เข็มกระตุ้น~~ที่สอง~~ และ 2.กลุ่มที่ไม่เคยมีการติดเชื้อมาก่อน (คัดกรองจากประวัติ)

สำหรับ secondary outcomes ทีมวิจัยจะแจกแจง percentage inhibition ของ sVNT ต่อ สายพันธุ์ที่กำลังมีการระบาดในขณะนั้นเช่น XBB, BA.2.75 และ delta, wild type virus และทำการเปรียบเทียบ percentage inhibition ทั้งสองกลุ่มโดยใช้ independent proportions test
และทำการประเมินความเกี่ยวข้องของ sVNT กับระดับ total anti-RBD IgG ในแต่ละกลุ่ม CD4 ด้วย

สุดท้ายจึงใช้ generalized linear models เพื่ออธิบายความสัมพันธ์ของ 1. การเพิ่มขึ้นระดับเม็ดเลือดขาวชนิดCD4 ในรูปแบบการเป็นตัวแปรต่อเนื่อง(continuous variable) 2. ระดับรวมของ anti-RBD IgG และ 3. sVNT percent inhibition หลังจากได้มีการปรับ confounder แล้ว

1. **ข้อพิจารณาด้านจริยธรรม (Ethical considerations)**

**หลักความเคารพในบุคคล (Respect for person)**
แพทย์ผู้ทำวิจัย อธิบายข้อมูลให้กับผู้ป่วยอย่างครบถ้วน และแจกเอกสารข้อมูลและแบบขอความยินยอมให้ผู้ป่วยพิจารณาก่อนตัดสินใจ เมื่อผู้ป่วยยินยอมแล้ว จึงถือว่าเข้าร่วมการวิจัยโดยผู้ป่วยสามารถขอยกเลิกการเข้าร่วมได้ ตลอด แม้ได้แสดงความยินยอมแล้ว ข้อมูลทั้งหมดของผู้ป่วยจะเป็นความลับ

**หลักการให้ประโยชน์ ไม่ก่อให้เกิดอันตราย (Beneficence/Non-maleficence)**

ผู้ป่วยได้รับการฉีดวัคซีนตามมาตราฐาน และได้ประโยชน์จากการกระตุ้นภูมิคุ้มกันจากวัคซีน และสังเกตอาการหลังฉีดตามมาตราฐานการเฝ้าระวังการฉีดวัคซีนโควิดของ CDC หากผู้ป่วยถูกประเมิณว่ามีผลข้างเคียงหลังจากการฉีดวัคซีน ผู้ป่วยจะได้รับการดูแล และบันทึกผลข้างเคียงหลังฉีดวัคซีนไว้ในเวชระเบียน

**หลักความยุติธรรม (Justice)**

**มี**เกณฑ์การคัดเข้าและออกชัดเจน หากระหว่างการติดตามผู้ป่วยติดเชื้อโควิด -19 โดยมีการทำ ATK ยืนยัน ผู้ป่วยจะถูกคัดออกจากการศึกษา รวมถึงได้รับการตรวจประเมิณความรุนแรงของโรค และพิจารณารับการรักษา

1. **ข้อจำกัดในการทำการวิจัย (limitation)**

การนัดเจาะเลือดซ้ำที่ 4 สัปดาห์ ซึ่งเป็นระยะเวลานานหลังจากฉีดวัคซีน อาจทำให้มีผู้ป่วย ไม่กลับมาเจาะเลือดตรวจติดตาม จำเป็นต้องมีโทรศัพท์ติดตามอาการของผู้ป่วยหลังจากฉีดวัคซีน และนัดหมายการมาตรวจเลือดซ้ำ

Sample size ที่ใช้มีปริมาณมากคือ ผู้ร่วมการศึกษา 122 คน ต้องอาศัยความร่วมมือในการรวบรวมผู้ป่วยจากคลินิกโรคติดเชื้อโรงพยาบาลจุฬา, ศูนย์วิจัยโรคเอดส์ สภากาชาดไทย และ HIV-NAT

1. **ผลหรือประโยชน์ที่คาดว่าจะได้รับจากการวิจัย (Expected Benefit and Application)**

ได้ทราบข้อมูลการตอบสนองทางแอนติบอดีภายหลังการฉีดวัคซีนโควิด-19 ชนิดmRNA เข็มกระตุ้น ในผู้ติดเชื้อเอชไอวีไทยที่มีระดับเม็ดเลือดขาวชนิดCD4 น้อยกว่าหรือเท่ากับ 200 เซลล์/มม.^3^ เทียบกับกลุ่มที่มี CD4 มากกว่า 200 เซลล์/มม.^3^ ในช่วงเวลาหลังการระบาด ที่คนส่วนใหญ่มีภูมิคุ้มกันทั้งจากวัคซีนและ การติดเชื้อตามธรรมชาติ
ซึ่งอาจให้ข้อมูลการตอบสนองที่ต่างจากการศึกษาเดิมที่เป็น primary vaccine และทราบความแตกต่างในเชิงปริมาณและคุณภาพ ของการตอบสนองทางแอนติบอดีหลังฉีดวัคซีน ระหว่างทั้งสองกลุ่ม CD4 หากการตอบสนองในกลุ่ม CD4 น้อยกว่าหรือเท่ากับ 200 เซลล์/มม.^3^ น้อยกว่าจริง อาจสามารถใช้เป็นข้อมูลเพื่อวางแนวทางการฉีดวัคซีนเข็มกระตุ้นในกลุ่มผู้ป่วยเอชไอวีในอนาคตได้

1. **อปุสรรคที่อาจเกิดขึ้นระหว่างการวิจัยและมาตราการในการแก้ไข(Obstacle)**

หากช่วงเวลาที่ทำการวิจัย มีการระบาดขอโควิด-19 อาจมีผู้เข้าร่วมอาจติดเชื้อระหว่างรอติดตามการเจาะเลือดครั้งที่สอง และทำให้ผู้เข้าร่วมต้องออกจากวิจัยเกิน 5% ทำให้ sample size ไม่ถึงที่คำนวณไว้ ทางทีมผู้วิจัยจะหาผู้เข้าร่วมเพิ่มในแต่ละกลุ่ม CD4 ที่ขาดหายเพื่อให้ถึงเกณฑ์จำนวน sample size

1. **การบริหารงานวิจัยและตารางการปฏิบัติงาน (Administration & Time Schedule)**

| กิจกรรม | พ.ศ. 2565 | | | | | | พ.ศ. 2566 | | | | | | | | | | | | | | | | | | พ.ศ. 2567 | | | | | | | | | | |  |
| --- | --- | --- | --- | --- | --- | --- | --- | --- | --- | --- | --- | --- | --- | --- | --- | --- | --- | --- | --- | --- | --- | --- | --- | --- | --- | --- | --- | --- | --- | --- | --- | --- | --- | --- | --- | --- |
|  | 7 | 8 | 9 | 10 | 11 | 12 | 1 | 2 | 3 | 4 | 5 | | 6 | 7 | | | 8 | 9 | | | 10 | 11 | | 12 | 1 | | 2 | 3 | | 4 | | 5 | | | 6 | |
| 1.ศึกษาเตรียมงาน | x | x | x | x | x | x | x | x | x | x | x | x | | |  |  | | |  |  | | |  |  |  |  | | |  | |  | |  |  | |  |
| 2.รวบรวมข้อมูล |  |  |  |  |  |  |  |  |  |  |  |  | | | x | x | | | x | x | | | x |  |  |  | | |  | |  | |  |  | |  |
| 3.วิเคราะห์ข้อมูล |  |  |  |  |  |  |  |  |  |  |  |  | | |  |  | | |  |  | | |  | x | x |  | | |  | |  | |  |  | |  |
| 4. รายงานผลการวิจัย |  |  |  |  |  |  |  |  |  |  |  |  | | |  |  | | |  |  | | |  |  |  | x | | | x | | x | | x | x | |  |

1. **งบประมาณ (Budget)**

คาดว่าวัคซีน mRNAเข็มกระตุ้นทั้งหมดจะได้รับการสนับสนุนจากทางรัฐบาล และ งบประมาณสำหรับตรวจการสนองทางภูมิคุ้มกัน และค่าตอบแทนสำหรับอาสาสมัคร มาจากทางสมาคมโรคเอดส์ แห่งประเทศไทย

| หมวดค่าใช้จ่าย | | รายละเอียด | งบประมาณ (บาท) |
| --- | --- | --- | --- |
| 1. | งบบุคลากร | - | 0 |
| 2. | งบดำเนินงาน   - ค่าตอบแทน - ค่าจ้าง - ค่าใช้สอย | -รวบรวม และวิเคราะห์ข้อมูล  -ค่าเดินทางอาสาสมัครผู้ป่วยโควิด-19 รวม 122 คน รวม 1 ครั้งๆละ 300 บาท  - ค่าบริการการตรวจ Anti RBD total Ig ตัวอย่างละ 500 บาท รวม 152 ตัวอย่าง | 10,000  36,600    122,000 |
| รวม (บาท) | | | 168,800 |

**ภาคผนวก dummy table**

| Characteristic | กลุ่ม CD4 น้อยกว่า 200 เซลล์/มม.^3^ | กลุ่ม CD4 มากกว่า 200 เซลล์/มม.^3^ | p-value |
| --- | --- | --- | --- |
| เพศ |  |  |  |
| อายุ, median |  |  |  |
| ระดับ CD4 ปัจจุบัน cells/mm^3^, median |  |  |  |
| เวลาตั้งแต่วินิจฉัย HIV (ปี), median |  |  |  |
| ประวัติ (virologic failure) (%) |  |  |  |
| สูตรของยาต้านไวรัส  RTIs, PIs, INSTIs (%) |  |  |  |
| ประวัติการฉีดวัคซีนโควิดก่อนหน้า  จำนวนเข็ม mRNA vaccine, median |  |  |  |

**เอกสารอ้างอิง (References)**

1. Bertagnolio S, Thwin SS, Silva R, et al. Clinical features of, and risk factors for, severe or fatal COVID-19 among people living with HIV admitted to hospital: analysis of data from the WHO Global Clinical Platform of COVID-19. Lancet HIV. In press.
2. TesorieroJM,SwainCE,PierceJL,etal.COVID-19 outcomes among persons living with or without diagnosed HIV infection in New York state. JAMA Netw Open **2021**; 4:e2037069.
3. Haidar G, Agha M, Bilderback A, et al. Prospective evaluation of COVID-19 vaccine responses across a broad spectrum of immunocompromising conditions: the COVICS study. Clin Infect Dis **2022**; 75:e630–44.
4. AntinoriA,CicaliniS,MeschiS,etal;HIV-VAC StudyGroup.Humoral and cellular immune response elicited by mRNA vaccination against SARS-CoV-2 in people living with HIV (PLWH) receiving antiretroviral therapy (ART) according with current CD4 T-lymphocyte count. Clin Infect Dis **2022**; 75:e552–63.
5. Hassold N, Brichler S, Ouedraogo E, et al. Impaired antibody response to
   COVID-19 vaccination in advanced HIV infection. AIDS **2022**; 36:F1–5.
6. Bhaskaran K, Rentsch CT, MacKenna B, et al. HIV infection and COVID-19 death: a population-based cohort analysis of UK primary care data and linked national death registrations within the OpenSAFELY platform. Lancet HIV **2021**; 8:e24–32.
7. FengY,ZhangY,HeZ,etal.Immunogenicity of an inactivatedSARS-CoV-2vaccine in people living with HIV-1: a non-randomized cohort study. EClinicalMedicine **2022**; 43:101226.
8. Xu X, Vesterbacka J, Aleman S, Nowak P; COVAXID Study Group. High sero- conversion rate after vaccination with mRNA BNT162b2 vaccine against SARS-CoV-2 among people with HIV—but HIV viremia matters? AIDS **2022**; 36:479–81.
9. Luo YR, Yun C, Chakraborty I, Wu AHB, Lynch KL. A SARS-CoV-2 Label-Free Surrogate Virus Neutralization Test and a Longitudinal Study of Antibody Characteristics in COVID-19 Patients. *J Clin Microbiol*. **2021**;59(7):e0019321.
10. Nie J, Li Q, Wu J, et al. Establishment and validation of a pseudovirus neutralization assay for SARS‐CoV‐2. Emerg Microbes Infect. **2020**;9(1):680‐686.
11. Focosi D, Maggi F, Mazzetti P, Pistello M. Viral infection neutralization tests: A focus on severe acute respiratory syndrome-coronavirus-2 with implications for convalescent plasma therapy. *Rev Med Virol*. **2021**;31(2):e2170.
12. Chalkias S, Harper C, Vrbicky K, et al. A Bivalent Omicron-Containing Booster Vaccine against Covid-19. N Engl J Med. **2022**;387(14):1279-1291.
13. Teeyapun N, Luangdilok S, Pakvisal N, et al. Immunogenicity of ChAdOx1-nCoV-19 vaccine in solid malignancy patients by treatment regimen versus healthy controls: A prospective, multicenter observational study. EClinicalMedicine. 2022;52:101608.
14. Chun HM, Milligan K, Agyemang E, et al. A Systematic Review of COVID-19 Vaccine Antibody Responses in People With HIV. Open Forum Infect Dis. 2022;9(11):ofac579.
15. Wang Q, Bowen A, Valdez R, et al. Antibody Response to Omicron BA.4-BA.5 Bivalent Booster [published online ahead of print, 2023 Jan 11]. *N Engl J Med*. 2023
16. Padoan A., Bonfante F., Pagliari M., Bortolami A., Negrini D., Zuin S., Bozzato D., Cosma C., Sciacovelli L., Plebani M. Analytical and clinical performances of five immunoassays for the detection of SARS-CoV-2 antibodies in comparison with neutralization activity. EBioMedicine. 2020;62:103101. doi: 10.1016/j.ebiom.2020.103101.
17. Claire Mullender, SARS-CoV-2 immunity and vaccine strategies in people with HIV, *Oxford Open Immunology*, Volume 3, Issue 1, 2022, iqac005
